# Middle meningeal artery embolization for chronic subdural hematoma: meta-analysis of three randomized controlled trials and review of ongoing trials

**DOI:** 10.1007/s00701-025-06587-4

**Published:** 2025-06-10

**Authors:** Conor S. Gillespie, Munashe Veremu, William H. Cook, Mohammad Ashraf, Keng Siang Lee, Youssef Chedid, Ali M. Alam, Yevgeny Karepov, Benjamin M. Davies, Ellie Edlmann, Panagiotis Papanagiotou, Stefanos Korfias, Thomas Santarius, Thais Minett, Peter J. Hutchinson, Angelos Kolias

**Affiliations:** 1 Department of Clinical Neurosciences, University of Cambridge, Downing Street, Cambridge, CB2 3EB UK; 2 Department of Neurosurgery, Cambridge University Hospitals NHS Foundation Trust, Cambridge, UK; 3 Wolfson School of Medicine, University of Glasgow, Glasgow, Scotland UK; 4 Department of Basic and Clinical Neurosciences, Maurice, Wohl Clinical Neuroscience Institute, Institute of Psychiatry, Psychology and Neuroscience , King’s College London, London, UK; 5 Institute of Infection, Veterinary and Ecological Sciences, University of Liverpool, Liverpool, UK; 6 Department of Radiology, Cambridge University Hospitals NHS Trust, Cambridge, UK; 7 Penninsula Medical School, Faculty of Health, University of Plymouth, Plymouth, UK; 8 Department of Diagnostic and Interventional Neuroradiology, Hospital Bremen-Mitte, Bremen, Germany; 9 Department of Radiology, Aretaieion University Hospital, National and Kapodistrian University of Athens, Athens, Greece; 10 Department of Neurosurgery, National & Kapodistrian University of Athens, Athens, Greece

**Keywords:** Middle meningeal artery embolization, MMAE, Chronic subdural hematoma

## Abstract

**Background:**

Middle Meningeal Artery Embolization (MMAE) has been proposed as adjunct and stand-alone treatment for Chronic Subdural Hematoma (CSDH). We aimed to meta-analyze three recently published randomized controlled trials, to reliably estimate the effect of MMAE. We also carried out a systematic review of ongoing trials and their key outcomes.

**Methods:**

A PRISMA-compliant meta-analysis was conducted (PROSPERO ID CRD42024618816). Three published RCTs (MAGIC-MT, EMBOLISE, and STEM) assessing MMAE in CSDH were included. Trial primary outcomes were pooled for analysis using random effects models. Primary and secondary outcomes (recurrence/surgical rescue, functional outcome) were obtained, stratified by treatment group (undergoing surgery, and nonsurgical management). A descriptive review of trials in public registries was also conducted (search date 30th November 2024).

**Results:**

In total, 1432 patients were included from three trials in meta-analysis. Overall, MMAE reduced symptomatic progression or recurrence, but was not statistically significant (RR 0.50, 95% CI 0.23–1.06, *P* = 0.058). For the group undergoing surgery, MMAE was not associated with reduced recurrence (RR 0.60, 95% CI 0.19–1.88, *P* = 0.194). For nonsurgical management, MMAE reduced progression (RR 0.36, 95% CI 0.22–0.60, *P* < 0.001). MMAE did not influence functional outcome (RR 1.01, 95% CI 0.97–1.04, *P* = 0.790). From the literature search, there are twenty-one registered trials. Nineteen studies include arms assessing MMAE as an adjunct to surgery, eleven compare MMAE to observation, and four with surgery. The most common primary outcome is recurrence (47.8%, *N* = 11), either radiologically, or requiring a second surgery. Inclusion criteria, embolization agents, primary and secondary outcomes differed significantly between studies.

**Conclusions:**

In this meta-analysis of three randomized controlled trials, the use of MMAE in patients undergoing surgery did not appear to significantly reduce recurrence or improve functional outcomes, but did reduce progression in nonsurgical cohorts. Further studies assessing these cohorts are ongoing.

**Supplementary Information:**

The online version contains supplementary material available at 10.1007/s00701-025-06587-4.

## Introduction

Chronic (nonacute) subdural hematoma (CSDH) is common, with an incidence of 14–20 per 100,000 people per-year [20] and is estimated to become the most common neurosurgical disease by 2030 due to a demographic shift towards an increasingly ageing population [46]. The increasing use of antithrombotic agents in the older population also contribute to the development of CSDH [3, 28]. Standard of care for patients with hematoma of large size, or progressive symptoms is surgical intervention, with burr-holes or craniotomy [4, 16, 28]. While surgical intervention is effective, recurrence ranges between 6 and 15% [15, 17, 18, 40, 50].

Middle Meningeal Artery embolization (MMAE) has been proposed as an additional treatment to reduce recurrence risk in CSDH, with increased reported utility in elderly patients on anti-platelet, anticoagulant medications, or with coagulopathies [1, 2]. Non-randomized retrospective and prospective studies have reported a reduced recurrence rate when used as both as an adjunct to surgery, and in nonsurgical management, with good overall outcomes [1, 7, 27, 33, 41]. However, while many nonrandomized studies exist [7, 14, 21, 43], this is confounded by a theoretical risk of treatment and selection bias. This precluded UK guidelines on CSDH, published earlier this year, from making a recommendation for MMAE in clinical care, instead proposing more research is required [45].

Three randomized controlled trials, have recently been published in the *New England Journal of Medicine (NEJM)* [10, 13, 31]. The effect of MMAE as i) an adjunct to surgery and ii) a standalone nonsurgical treatment, was assessed by the MAGIC-MT (Managing Non-acute Subdural Hematoma Using Liquid Materials: a Chinese Randomized Trial of Middle Meningeal Artery Treatment [31]; and the STEM (Squid Trial for the Embolization of the Middle Meningeal Artery for the Treatment of Chronic Subdural Hematoma) trials [13]. The EMBOLISE (Embolization of the Middle Meningeal Artery with Onyx Liquid Embolic System in the Treatment of Subacute and Chronic Subdural Hematoma) [10] study assessed MMAE as adjunctive to surgery in comparison with surgery alone. All studies reported a lower risk of recurrence or progression with MMAE, compared to control groups, although in MAGIC-MT this difference was not significant [24, 31].

As the trials share a similar patient cohort, the same intervention, and a similar primary outcome (clinical/radiological recurrence or progression) a combined meta-analysis would be helpful in evaluating the overall treatment effect of MMAE. The trials also included both patients with CSDH undergoing surgery and nonsurgical management, but combined their overall results. It is thus pertinent to obtain an estimate for the effect of MMAE as an adjunct in those undergoing surgery, in addition to MMAE for CSDH managed nonsurgically. This article performs a meta-analysis of Level 1 evidence, with the primary aim of estimating the effect of MMAE based on pooled data from all of the trials, as an adjunct to surgery, and in non-surgically managed CSDH.

We also carried out a review of ongoing trials in MMAE, to establish other studies being conducted worldwide, their key inclusion criteria, and choice of outcomes.

## Methods

### Meta-analysis

We included multi-center, published randomized control trials. Hence, we combined overall study data from three recently published trials- MAGIC-MT (NCT04700345), EMBOLISE (NCT04402632), and STEM (NCT04410146), which were all open-label, randomized, controlled clinical trials assessing the effect of MMAE in patients with CSDH. The trials were all published on 20 th November 2024 [10, 13, 31]. In summary, MAGIC-MT included 722 patients with symptomatic CSDH, randomized to receive MMAE versus usual care after their surgeon had decided if they will be managed surgically or non-operatively. EMBOLISE included 400 patients with symptomatic CSDH undergoing surgery randomized to addition of MMAE or usual care. STEM included 310 patients with symptomatic CSDH, randomized to receive MMAE in addition to best usual management (including observation alone or surgery) versus usual management alone.

Randomization, treatment, and outcome assessment was done according to the individual study protocols, and approved by relevant institutional boards. Inclusion and exclusion criteria for the three trials were similar, with all patients recruited having symptomatic CSDH, and an initial treatment decision made by treating clinicians before randomization, to either surgery or conservative management.

The primary outcomes were defined as follows:

- In the MAGIC-MT trial (Onyx™); symptomatic recurrence or progression of the subdural hematoma (maximum thickness > 10 mm) within 90 days of randomization.
- In EMBOLISE (Onyx™); recurrence or progression that led to repeat surgery within 90 days after index treatment.
- In STEM (Squid®); composite of recurrent or residual hematoma, surgical rescue, or major disabling stroke, myocardial infarction, or death within 180 days of randomization.

The recurrence definitions were similar between studies. In summary, MAGIC-MT defined recurrence as maximum hematoma thickness of > 10 mm or reoperation with burr hole drainage, or progression of more than 3 mm from baseline or surgical rescue for those treated only with MMAE. EMBOLISE defined recurrence as imaging evidence of subdural hematoma with or without worsening symptoms, that required repeat surgery. STEM defined recurrence as recurrent or residual hematoma of > 10 mm, or surgical rescue. Secondary outcomes varied between studies, but in short, MAGIC-MT collected recurrence at 1 year, change in hematoma thickness, and modified Rankin Scale (mRS) scores at 90 days, 1 year, and the change between them, in addition to serious adverse events and complications. EMBOLISE collected neurological function at 90 days (mRS), and changes from baseline, as well as safety endpoints, hospitalization data, and hematoma radiological outcomes. STEM collected mRS, adverse outcomes and serious events at 180 days.

In the MAGIC-MT trial, the primary outcome (symptomatic recurrence or progression of the subdural hematoma) occurred in 6.7% of the MMAE group, and 9.9% in control group (*P* = 0.100). In EMBOLISE, the primary outcome (recurrence or progression that led to repeat surgery) occurred in 4.1% of the MMAE group, and 11.3% in the control group (*P* = 0.008). In STEM, the primary outcome (composite of recurrent or residual hematoma, surgical rescue, or major disabling stroke, myocardial infarction, or death) occurred in 16% of the MMAE group and 36% in the control group (*P* = 0.001).

### Procedures and data extraction

All data was extracted from the full published trial manuscripts. Baseline demographics collected included trial name, population, allocation size, treatment, embolization material, and follow-up period. For the meta-analysis, we used the primary outcome defined by the authors. We collected the overall study data (including all patients), and sub-grouped data specific for those undergoing surgery plus MMAE (or surgery alone as the control group), or observation plus MMAE (or observation alone as the control group). In addition, we collected 90-day reoperation rates for patients undergoing surgery plus MMAE (or surgery alone as the control group). We also collected functional outcomes, which were defined as a secondary endpoint, measured by the mRS in all three trials. The mRS dichotomization varied between studies, with the MAGIC-MT trial defining a score of 0–2 as ‘good’ and a 0–3 as ‘favourable’. EMBOLISE presented scores of 0–2, and 0–3, but did not define them. The STEM trial presented median scores for mRS, and a comparison of differences pre and post-surgery between groups.

### Statistical analysis

For the meta-analysis, we used a random effects model of binary outcomes for the primary outcome (recurrence or rescue surgery), in accordance with previously published methods [29]. We generated forest plots based on a random intercept model, using inverse variance method, and by applying a Hartung-Knapp adjustment for meta-analysis including less than 20 studies to improve accuracy [19, 49]. For each random effects model, we tested heterogeneity using the restricted maximum likelihood (REML) method. Total heterogeneity and I^2^ characteristics were calculated. As only three trials are included, publication bias was not formally assessed, but funnel plots were included for completeness.

We first carried out an analysis for overall primary endpoint (recurrence or progression) with all study data available from all three trials. We then carried out an analysis examining the sub-groups; surgery ± MMAE and nonsurgical ± MMAE. For the primary outcome, the GRADE framework was used to assess the certainty of evidence.

We also assessed the impact of MMAE on mRS, where included. The meta-analysis was performed utilizing R statistics (RStudio Version 4.0.1), including creation of figures, and forest plots (ggplot2 and meta packages). We carried out a sensitivity analysis, excluding the STEM trial, which had a longer study endpoint (180 days). We also included fixed effects models in sensitivity analysis, in addition to random effects analysis with the Mantel–Haenszel method. Risk of bias assessment was not performed, as the studies have recently been published. We considered a P value < 0.05 as significant. For consistency, analysis was also repeated using RevMan version 7.2.0 [9].

### Trials review- search strategy and selection criteria

We conducted a systematic review of ongoing trials according to the Preferred Reporting Items for Systematic Reviews and Meta-Analyses (PRISMA) guidelines (PROSPERO CRID CRD42024618816) [36]. We searched nineteen trial databases (Supplementary Table 1). The databases were searched from inception to 15 th May 2024, and updated on 30th November 2024. Search terms are shown in Supplementary Table 2. Population, Intervention, Comparator, Outcome, and Study Design (PICOS) criteria were used (Table 1).

**Table 1:** Population, intervention, comparator, outcome, study design (PICOS) criteria used for the review

Review question	What are the current ongoing trials investigating Middle Meningeal Artery Embolization for Chronic Subdural Hematoma, and what are the main outcome measures?
Population	Adults ≥ 18 years with CSDH
Intervention	Either: • MMAE alone • Surgery + MMAE
Comparator	Either: • Surgery alone • Conservative management/best medical care
Outcomes	Primary: • Identify primary outcome used in trials • Identify definitions used in ongoing trials Secondary: • Identify the number of ongoing trials • Identify methodologies (including trial inclusion and exclusion criteria)
Setting	Studies taking place in any neurosurgery, trauma or radiology/neurology department or center
Study design	Must be a randomized trial, with an intervention and control group- exclude prospective cohort studies or studies with a single arm Include studies regardless of blinding, as long as there is a random allocation to at least two groups
Follow-up	Any follow-up

### Data extraction

Two reviewers (CSG, MV) screened articles, and completed data extraction, with the senior author (AK) making a final determination in any conflicts. We gathered data on trial status, sponsors, estimated completion date, and study specific data, such as embolic agent, intervention definition, follow up period, and primary and secondary outcomes. Quality assessment was not completed as part of the study, as the aim was to identify ongoing trials for descriptive purposes.

## Results

### Meta-analysis

In total, 1432 participants were included from the three trials (722 MAGIC-MT, 400 EMBOLISE, and 310 STEM). The surgical cohort had 1114 patients with the primary outcome available (550 with MMAE and surgery, 564 with surgery alone), and in the nonsurgical cohort, 257 patients had the primary outcome available (127 MMAE alone, 130 nonsurgical alone). As the trials did not contain individual data, it was not possible to pool baseline characteristics. In MAGIC-MT, the median age was 69 years, with 82.5% male, and 7.3% taking antithrombotic medication. In EMBOLISE, the mean age was 72 years, with 73.0% male patients, and 38.5% taking antithrombotic medication. In STEM, the mean age was 73 years, with 70% male patients, and 40% taking antithrombotic medication.

**Fig. 1:**
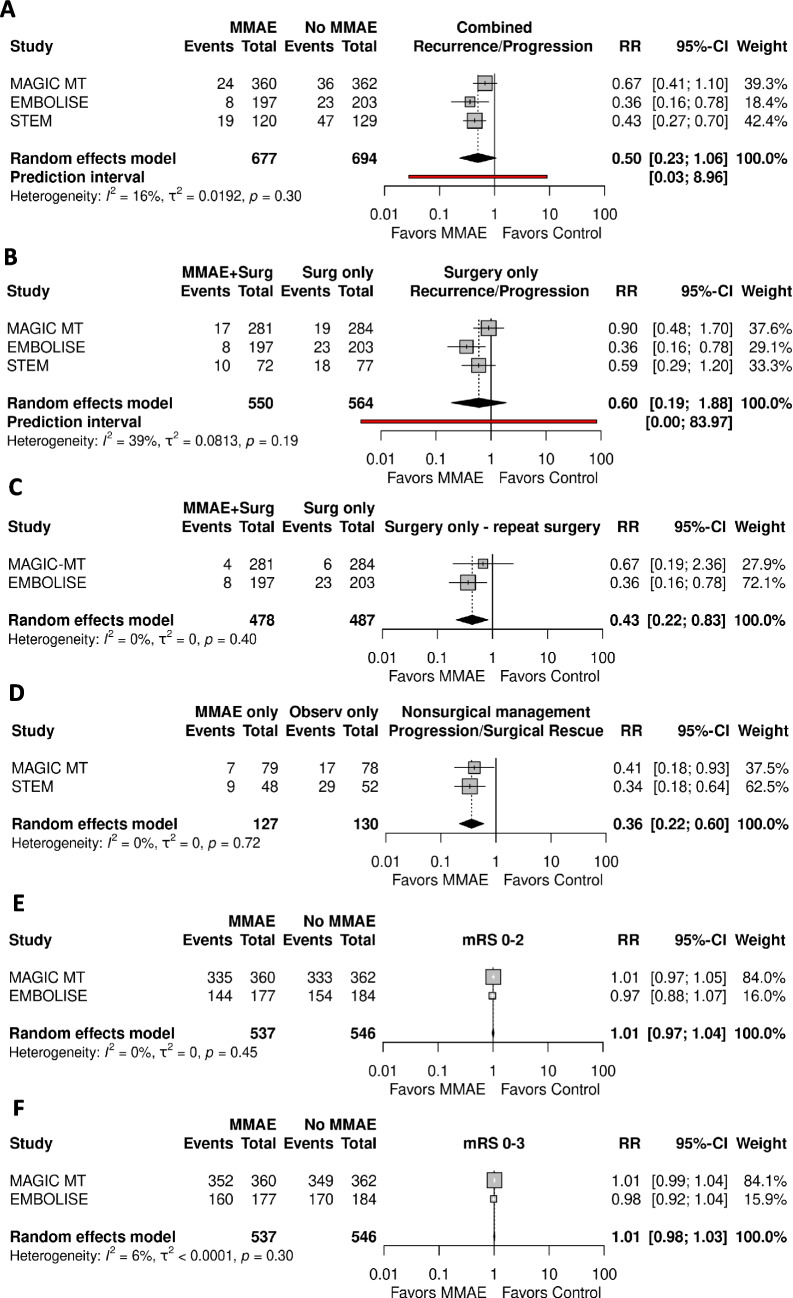
Forest plot of **A**) Meta-analysis from three studies for primary outcome (progression/recurrence), comparing MMAE and usual care (i.e. surgery or non-surgical); **B**) Primary outcome in patients undergoing MMAE + surgery and surgery only; **C**) Primary outcome of repeat surgery at 90 days in patients undergoing MMAE + surgery and surgery only **D**) Primary outcome in patients undergoing nonsurgical management only; **E**) Modified Rankin Scale 0–2 comparing MMAE and usual care **F**) Modified Rankin Scale 0–3 (at follow-up comparing MMAE and usual care

Figure 1 shows the results of the meta-analysis. Overall, use of MMA was associated with reduced risk of recurrence, but was not statistically significant (RR 0.50, 95% CI 0.23–1.06, I^2^ = 16%, *p* = 0.058).

When examining the group that underwent surgery, MMAE did not significantly reduce recurrence (RR 0.60, 95% CI 0.19–1.88, I^2^ = 39%, *p* = 0.194). When selecting re-operation at 90 days as the outcome of interest, MMAE in addition to surgery did reduce reoperation rate (RR 0.43, 95% CI 0.22–0.83, I^2^ = 0%, *p* = 0.012).

When examining only the non-operative cohort, MMAE was statistically significant at preventing rescue surgery/progression (RR 0.36, 95% CI 0.22–0.60, I^2^ = 0%, *p* < 0.001).

For functional outcome, two trials (MAGIC-MT, EMBOLISE) included the mRS breakdown for meta-analysis. There was no difference in MMAE and control groups in achieving mRS 0–2 (RR 1.01, 95% CI 0.97–1.04, I^2^ = 0%, *p* = 0.7905) or mRS 0–3 (RR 1.01, 95% CI 0.99–1.03, I^2^ = 6%, *p* = 0.443) at 90 and 180 days. The sensitivity analysis is shown in Supplementary Figs. 1, 2, 3, 4, 5, 6 and 7. Funnel plots for all outcomes are provided in Supplementary Figs. 8,9,10, 11, 12 and 13.

Using fixed effects models, the odds ratios were similar to random effects. For fixed effects models, MMAE was associated with reduced occurrence of primary outcome when all patient cohorts were combined (RR 0.50, 95% CI 0.37–0.68, p < 0.001), in patients managed with surgery (RR 0.60, 95% CI 0.41–0.90, p < 0.001), and nonsurgical management (RR 0.36, 95% CI 0.22–0.60, I^2^ = 0%, p < 0.001). Removing data from the STEM trial (180-day follow-up) produced similar results. Using Mantel–Haenszel modelling produced similar results (Supplementary Figs. 14, 15, 16, 17, 18 and 19). Using RevMan produced identical results (Supplementary Figs. 20, 21, 22, 23 and 24). The certainty of evidence according to the GRADE framework was assessed as ‘moderate’ due to indirectness (Supplementary Table 5).

### Systematic review and characteristics

**Fig. 2:**
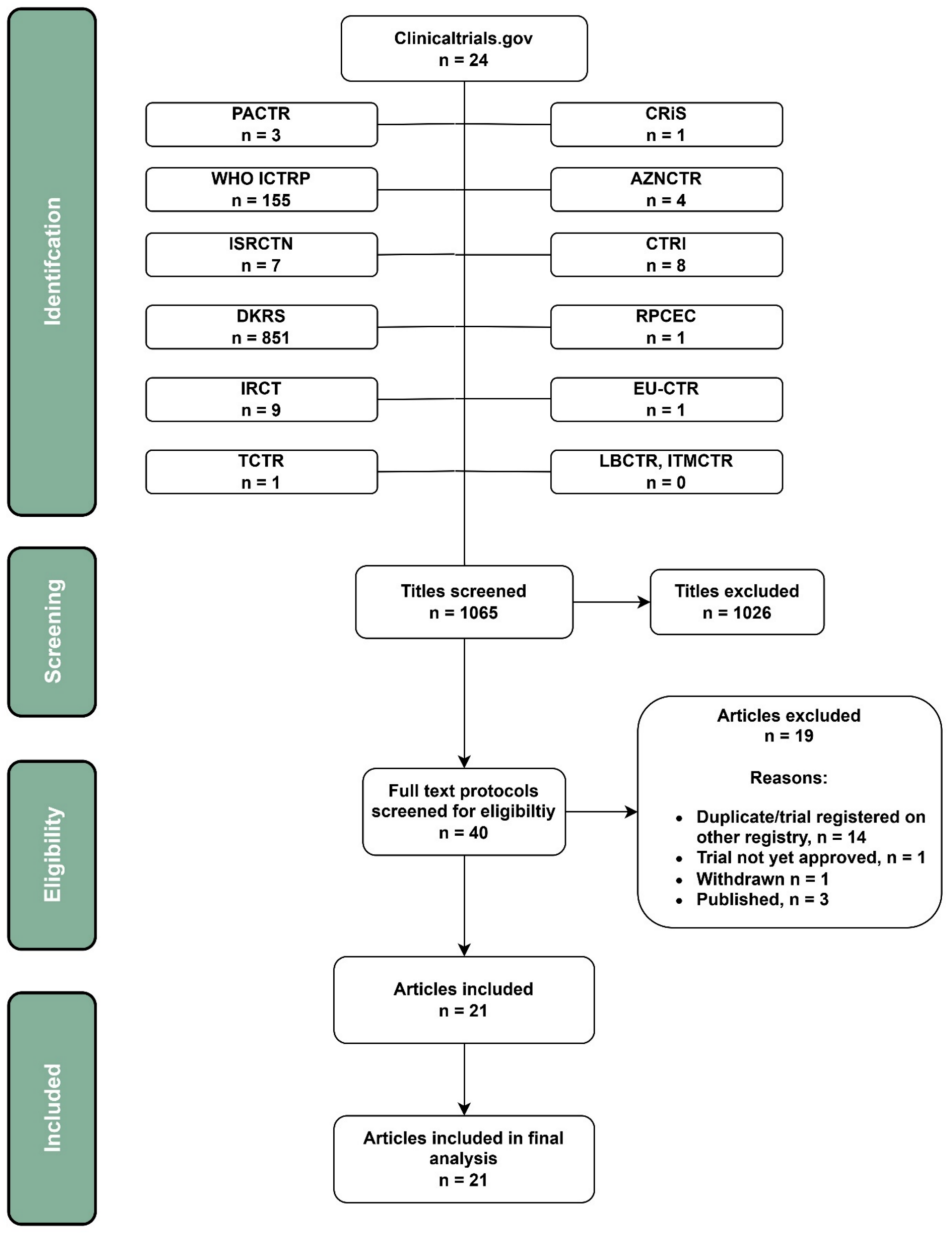
PRISMA flow-chart

After full-text assessment, 21 total protocols were included (supplementary Table 3), (Fig. 2).

### Trial characteristics

**Fig. 3:**
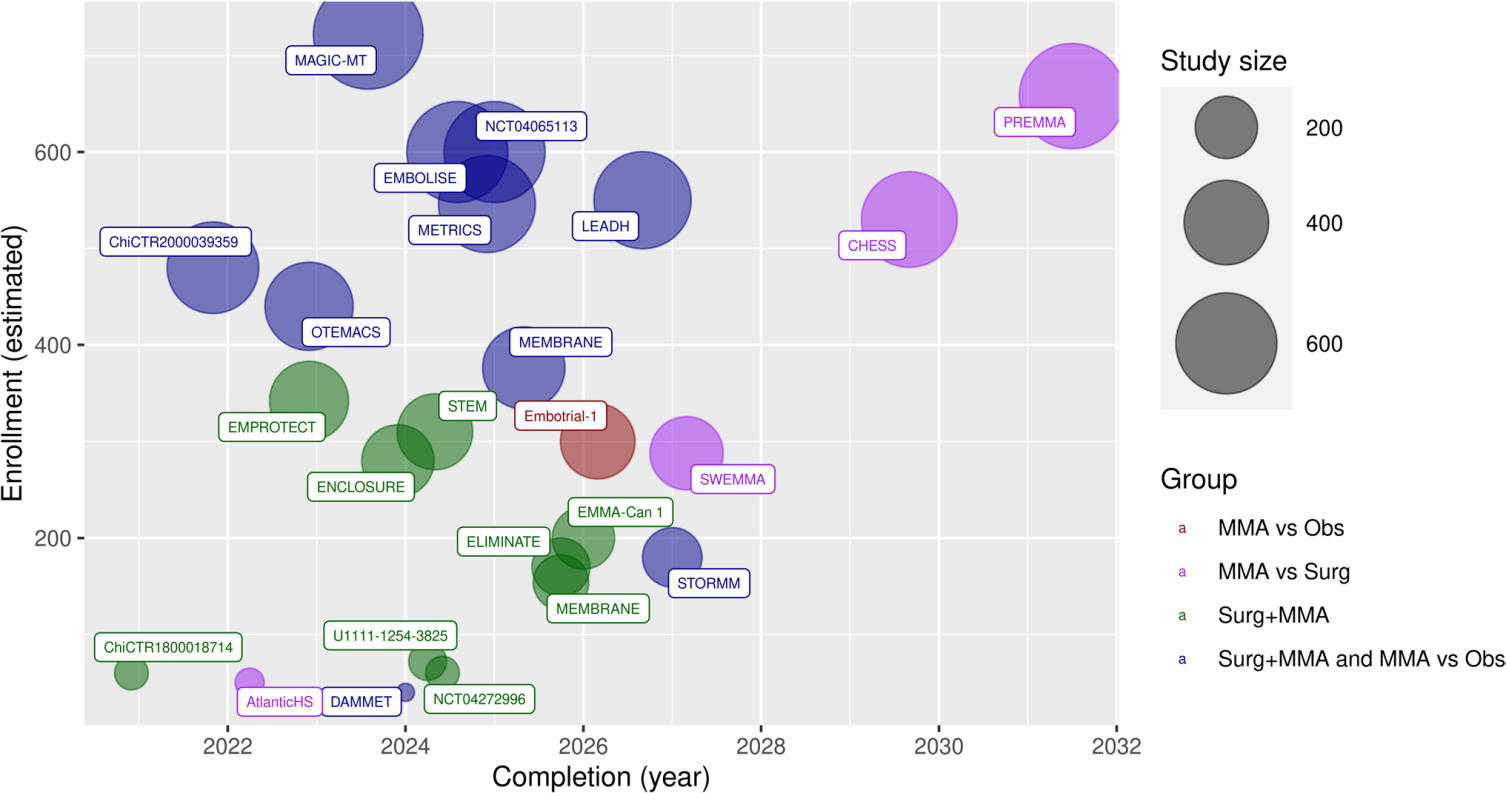
Bubble plot of ongoing and recently published MMA trials, grouped by trial design/arms. Bubble size = reflective of total population size of trial. *MMA vs Obs = Trial of MMAE Vs nonsurgical management only; MMA vs Surg = Trial of MMAE alone vs surgery; Surg + MMA = Trial of MMAE + Surgery vs Surgery; Surg + MMA and MMA vs Obs = Trial including both arms

The trial characteristics are shown in Table 2. Most trials were active and recruiting (62%, *N* = 13). Approximately half of studies required patients to be symptomatic to be included (52%, *N* = 12). About half of the studies included patients with a specific baseline performance status (48%, *N* = 10). Eight studies (38%) included four arms- surgery versus surgery plus MMAE, and observation versus MMAE alone. Eight studies (38%) only assessed surgery versus surgery plus MMAE. Four trials are assessing MMAE versus surgery (19%), with one assessing MMAE vs observation. The trial categories, estimated population size, and completion dates are shown in Fig. 3. Most did not mention the embolization agent to be used (41%, *N* = 7). The most commonly listed agent was Onyx ™ (12%, *N* = 2). The range of agents included were “Particles or Liquid embolic agents”, Embosphere®, PVA particles, Squid®, cyanoacrilates, Onyx™, Phil™, and Libro®.

**Table 2:** Summary of included studies’ protocol characteristics

Characteristic	Value (%)
Included studies	21
Clinicaltrials.gov	17 (81)
ICTRP	3 (14)
AZNCTR	1 (5)
Location	Value (%)
USA	6 (29)
China	3 (14)
France	3 (14)
Other	9 (43)
Status (1/12)	Value (%)
Recruiting	12 (57)
Not yet recruiting	4 (19)
Unknown	2 (10)
Active, not recruiting	1 (5)
Completed	2 (10)
Year Registered	Value (%)
2018	1 (5)
2019	2 (10)
2020	6 (29)
2021	4 (19)
2022	3 (14)
2023/2024	5 (24)
Sponsor type	Value (%)
Hospital/University	17 (85)
Industry	1 (5)
NIH	1 (5)
Hospital and Industry	2 (10)
Mean estimated enrollment (SD) [Range]	304 (202) [40–658]
Full study protocol published?	2 (10)
Inclusion criteria	Value (%)
CSDH + symptoms	11 (52)
CSDH + radiological criteria	7 (33)
CSDH + performance status	10 (48)
mRS 3 or less	5 (50)
mRS 4 or less	2 (20)
mRS 2 or less	3 (30)
Trial type	Value (%)
Surgery plus MMA	16 (76)
MMA vs observation	9 (43)
MMA vs surgery	4 (19)
Trial groups	
Both of: Surgery + MMAE vs surgery only Non-operative: Conservative management + MMAE vs conservative management only	8 (38)
Surgery + MMAE vs Surgery only	8 (38)
Surgery vs MMAE only	4 (19)
MMAE vs Observation only	1 (5)
Follow-up and primary outcomes	Value (%)
6 months	11 (53)
3 months	6 (29)
12 months	2 (10)
Not stated	2 (10)
Primary outcome	Value (%)
Radiological recurrence or recurrence requiring surgery	9 (43)
Requiring surgery only	6 (29)
Radiological only	4 (19)
Not defined	2 (10)

### Trials assessing MMA as an adjunct to surgery

In total, 16 trials have arms that assess MMAE as an adjunct to surgery (surgery plus MMAE vs surgery only). These are shown in Table 3. The most commonly used agent is PVA (*N* = 4) followed by Onyx™ (*N* = 3). The most common follow-up period was 6 months (*N* = 8), and the most common primary outcome was radiological recurrence or recurrence requiring repeat surgery (*N* = 8). MEMBRANE and EMPROTECT results have been referenced in published studies, with no full trial results published currently [8].

**Table 3:** Trials assessing MMAE as adjunct to surgery (*N* = 16)

Trial Name	Country	Size	End date	Status	Population	Industry funded	Agent	Primary outcome	Follow-up
DAMMET	USA	40	01–24	Completed	None	No	Not stated	Repeat surgery	3 months
STORMM	Switzerland	180	01–27	Not yet recruiting	Symptoms or failed conservative	No	Not stated	Symptomatic recurrence, repeat surgery, or on scan	6 months
OTEMACS	France	440	12–22	Unknown	Symptoms + MRS 3 or less	No	Onyx™	Repeat surgery or radiological	3 months
MEMBRANE	Germany	376	05–25	Active, not recruiting	MRS 3 or less	Yes	TRUFIILL n-BCA® (Cyanoacrilates)	Resolution	6 months
LEADH	France	550	09–26	Recruiting	None	No	Cyanoacrilates	Repeat surgery or radiological	6 months
ChiCTR2000039359	China	480	11–21	Not yet recruiting	“Requiring surgery”	No	Not stated	Repeat surgery or radiological	Not stated
METRICS	China	546	12–24	Not yet recruiting	One risk factor for recurrence	No	Not stated	Repeat surgery or radiological or new disability from neurological cause	6 months
NCT04065113	USA	600	01–25	Unknown	Symptoms	No	PVA particles	Repeat surgery	3 months
ELIMINATE	Netherlands	170	10–25	Recruiting	Symptoms	No	PVA particles	Repeat surgery	6 Months
EMMA-Can 1	Canada	200	01–26	Recruiting	Symptoms and MRS 2 or less	No	PVA particles	Radiological	3 Months
MEMBRANE	Germany	154	10–25	Recruiting	None	No	PVA particles or Onyx™	Repeat surgery or radiological	3 Months
ENCLOSURE	Spain	280	12–23	Unknown	> 10 mm or symptoms	No	Onyx™, SQUID®, Phil™, Libro®	Radiological	6 Months
EMPROTECT	France	342	12–22	Unknown	Recurrence or high risk of	No	Not stated	Repeat surgery or radiological	6 Months
ChiCTR1800018714	China	60	12–20	Recruiting	Symptoms	No	Not stated	Repeat surgery	Not stated
NCT04272996	USA	60	06–24	Recruiting	Symptoms and MRS 4 or less	No	Not stated	Radiological	3 Months
U1111-1254–3825	Australia	72	04–24	Recruiting	Symptoms	Yes	Not stated	Radiological and new symptoms requiring surgery	6 Months

### Trials assessing MMAE compared to best medical care and observation

In total, 9 trials have arms that assess MMAE compared to best medical management and observation (Table 4). The most commonly used agent is PVA particles (*N* = 2). The most common follow-up period is 6 months (*N* = 5), and the most common primary outcome is radiological recurrence or recurrence requiring repeat surgery (*N* = 5).

**Table 4:** Trials assessing MMAE compared to best medical care in conservatively managed CSDH (*N* = 9)

Name	Country	Size	End date	Status	Population	Industry funded	Agent	Primary outcome (Observation groups)	Timepoint
DAMMET	USA	40	01–24	Completed	None	No	Not stated	Repeat surgery	3 months
STORMM	Switzerland	180	01–27	Not yet recruiting	Symptoms or failed conservative mx	No	Not stated	Symptomatic recurrence, repeat surgery, or on scan	6 months
OTEMACS	France	440	12–22	Unknown	Symptoms + MRS 3 or less	No	Onyx™	Repeat surgery or radiological	3 months
MEMBRANE	Germany	376	05–25	Active, not recruiting	MRS 3 or less	Yes	TRUFIILL n-BCA (Cyanoacrilates)	Progression or requiring re-intervention	6 months
LEADH	France	550	09–26	Recruiting	None	No	Cyanoacrilates	Repeat surgery or radiological	6 months
ChiCTR2000039359	China	480	11–21	Not yet recruiting	“Requiring surgery”	No	Not stated	Repeat surgery or radiological	Not stated
METRICS	China	546	12–24	Not yet recruiting	One risk factor for recurrence	No	Not stated	Repeat surgery or radiological or new disability from neurological cause	6 months
NCT04065113	USA	600	01–25	Unknown	Symptoms	No	PVA particles	Repeat surgery or radiological	3 months
Embotrial-1	Italy	300	03–26	Not yet recruiting	No symptoms, MRS 2 or less	No	PVA particles or liquid embolic materials	Requires surgery or hematoma resolution	6 months

### Trials assessing MMAE vs surgery

In total, four trials registered aim to assess MMAE directly compared to surgery (Table 5). Repeat surgery was the primary outcome used in three studies, with radiological resolution of hematoma the other.

**Table 5:** Summary of trials examining Surgery vs MMAE (*N* = 4)

Name	Country	Size	End date	Status	Population	Industry funded	Agent	Primary outcome	Endpoint
CHESS	USA	530	09–29	Not yet recruiting	None	No	EmboSphere® or PVA particles	Repeat surgery	6 months
AtlanticHS	USA	50	04–22	Unknown	Symptoms	No	Not listed	Hematoma resolution	6 months
SWEMMA	Sweden	288	03–27	Recruiting	Markwalder score < 2 + GCS > 13	No	Not listed	Repeat surgery	3 months
PREMMA	Puerto Rico	658	07–31	Not yet recruiting	Age ≥ 21, markwalder score ≤ 2 + GCS ≥ 14, resident in Puerto Rico	No	PVA particles	Repeat surgery	3, 6, and 12 months

### Follow-up and primary outcomes

The most common follow-up period for all included trials is 6 months (43%, *N* = 9). The most common primary outcome was recurrence, either radiologically, or requiring a second operation/surgery (43%, *N* = 9). The secondary outcome measures are shown in Supplementary Table 4. The most common secondary outcomes assessed were mRS (62%, *N* = 13), hematoma resolution (48%, *N* = 10), and mortality (38%, *N* = 8).

## Discussion

### Key findings

This meta-analysis of three published randomized control trials confirms suggestions from non-randomized studies, that MMAE is safe and has a role to play in CSDH for reducing progression and/or recurrence. However, in the meta-analysis, the effect size failed to reach statistical significance. MMAE did not improve primary outcomes in surgical patients, but did reduce progression in nonsurgical patients. MMAE did not result in more favorable functional outcomes compared to conventional management [24]. The review of ongoing trials identified they have heterogeneous recurrence definitions, primary outcomes, and do not include cost-effectiveness in their planned analysis.

All trials included in the meta-analysis were randomized, controlled, and had broadly similar primary outcomes of radiological recurrence or recurrence/progression, although the STEM trial also included stroke, myocardial infarction, or death from neurological cause. Whereas several meta-analyses have been produced for non-randomized studies [21, 30], our study reports on a meta-analysis of randomized control trials, assessing the highest level of evidence, thereby minimizing bias as much as possible. Two trials had significant loss to follow-up (EMBOLISE, 13.2%, and STEM, 15% respectively), which is important to note when assessing outcomes.

**Fig. 4:**
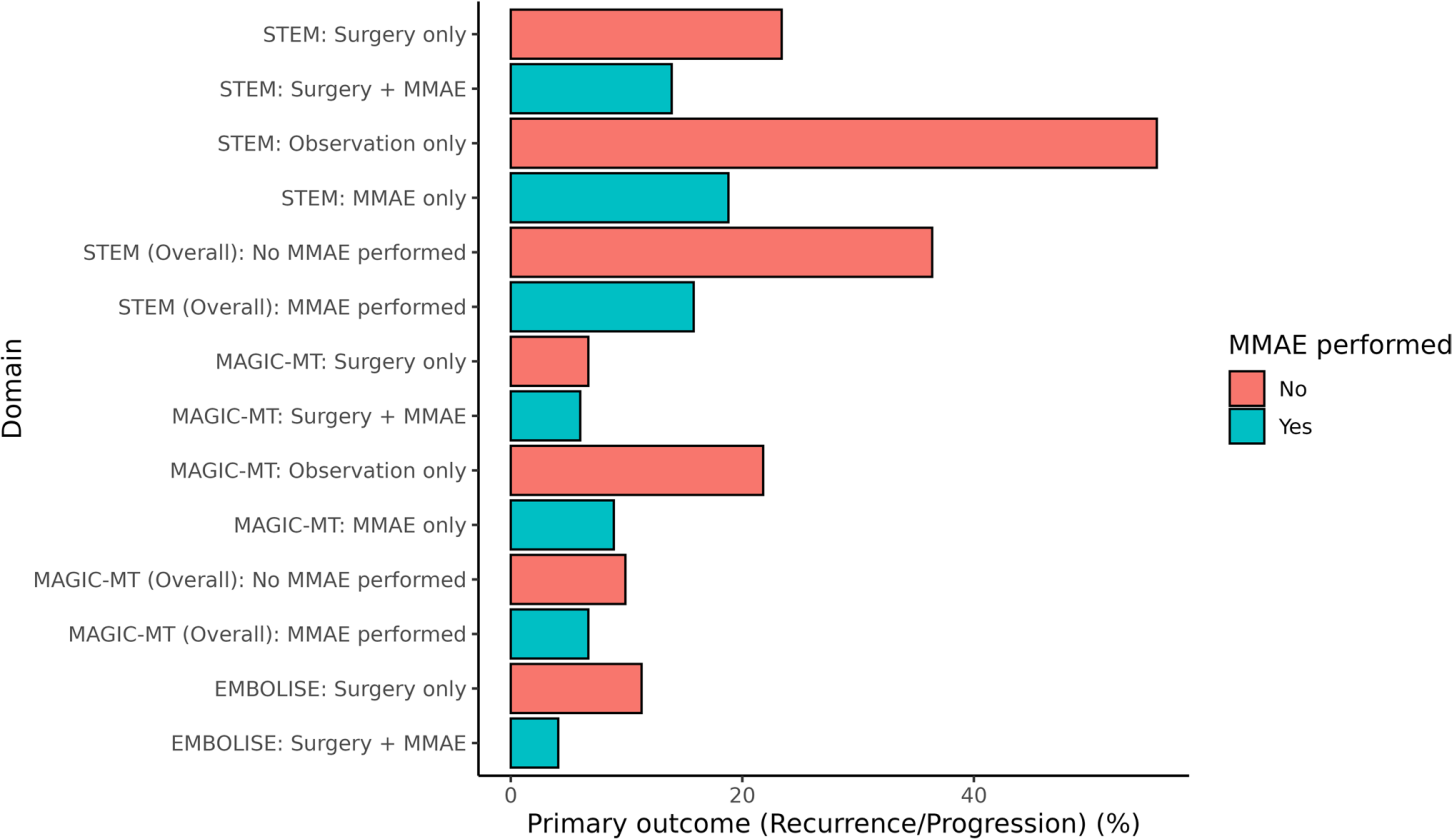
Stacked bar chart demonstrating differences in primary outcome MMAE groups (red) compared to non-MMAE groups as controls (blue), stratified by cohort (overall, surgical, and nonsurgical)

Both MAGIC-MT and STEM trials combined non-surgical and surgery cohorts in their analysis of MMAE effectiveness. While all trials, except MAGIC-MT reported statistically significant differences, in MAGIC-MT and STEM, MMAE was far more effective in those with nonsurgical management. In MAGIC-MT, progression in the nonsurgical management cohort was 8.9% in the treatment compared to 21.8% in the control groups. A similar result was seen in STEM (18.8% treatment vs. 55.8% control). In the surgery only cohort, these differences were less pronounced (MAGIC-MT 6.0% MMAE versus 6.7% control, STEM 13.9% MMAE versus 23.4% control). This indicates that the differences seen in the trial data, may largely be driven by MMAE effectiveness in the nonsurgical cohort, and not those managed with surgery. On the other hand, the only trial that examined the role of MMAE as an adjunct to surgery (EMBOLISE) had a highly significant result. These differences are illustrated in Fig. 4.

Two trials used 90 days as the minimum follow up period with the STEM trial using 180 days as the trial endpoint. This may explain why the STEM trial had the highest progression or recurrence rate among the trials, in addition to including stroke, myocardial infarction, or death in their composite outcome measure. All trials included radiological definitions of progression or recurrence in their primary outcome measure, although this differed for each trial.

Neither trial included cost-effectiveness analysis, with all three trials receiving funding by the embolic agent manufacturer. None of the planned or ongoing trials included plans for cost-effectiveness analysis. Previous retrospective studies have provided data to both support and refute the cost-effectiveness of MMAE. Two propensity matched studies have identified that MMAE is significantly more expensive to perform than conventional surgery [26, 39, 47], with one study reporting over three times as expensive [39]. One study suggested that at one year, MMAE was more cost-effective when taking into account reduced recurrence rates and additional costs [6]. Furthermore, MMAE could potentially reduce hospital length of stay [5, 34], by reducing the need of hematoma re-treatment, and derived complications from this such as seizures, empyema, and hospital acquired infections [37].

There have been numerous published systematic reviews and meta-analysis on MMAE in CSDH, with many using the same number of studies [21, 43]. Treatment bias has been identified as a significant limitation in several reviews that highlight the promise of MMAE [7]. This overlaps with the RCT findings, which showcase a disproportionate benefit among nonsurgically managed patients- and also the findings of a recent systematic review [38]. Other studies have been produced concluding MMAE is a safe and effective approach for CSDH, with one using conference abstract data, and using different methods of meta-analysis, which explains the difference in results with our study [8, 11, 23]. Of note, we used inverse variance methods, and applied a Hartung-Knapp adjustment, which is generally recommended [22, 29, 49].

MMAE has not been directly compared to surgery in any of the published RCTs. One retrospective study of 142 participants has evaluated failure rates in MMAE alone compared to surgical groups, and identified a comparable treatment failure rate between groups, but an earlier rate of neurological deterioration requiring surgery in the MMAE group [42]. International guidelines have made provisional recommendations for the use of MMAE, but this will certainly change following the publication of the randomized controlled trials [2, 25, 35].

### Implications for future trials of MMAE

We believe there are unanswered research questions in MMAE for CSDH. Firstly, the cost-effectiveness of MMAE is uncertain, particularly in those undergoing surgery, where the benefits appear to be less clear. Secondly, the ideal choice of embolic agent is unknown. Multiple different types are being used in trials, but no trial explicitly is seeking to compare the agents. Particles, coils and liquid embolic agents have different profiles in terms of safety, durability, cost, need for general anesthetic, and potentially efficacy. A recent review appraised many of these agents, and should MMAE become increasingly common practice [48], the outcomes, complications and cost-effectiveness of all potential treatment modalities should be examined. While independent from the study group, two trial manuscripts were drafted by, or had statistical analysis assisted by the manufacturer who supplied the embolic agent. Thirdly, its application to all CSDH patients requires consideration. The STEM and MAGIC-MT trials notably selected patients with a better baseline (mRS < 2), with STEM including mRS 0–1, and MAGIC-MT mRS 0–2, which represents < 75% of CSDH patients treated in routine care [4, 44]. Fourth, the benefit of MMAE for patients with a higher risk of recurrence who require the use of anti-thrombotic agents for other co-morbidities remains unknown. The risks of holding the anti-thrombotic medications cannot be underestimated ranging from stroke, myocardial infarct, pulmonary embolism and others. MMAE could potentially reduce the time without such medications, preventing complications [2].

Finally, and perhaps most importantly, questions remain around the use of recurrence as the primacy outcome. Notwithstanding significant heterogeneity in its definition (whether this be hematoma resolution only, radiological recurrence, radiological recurrence or surgery, or recurrence requiring surgery) recurrence by any definition is a surrogate outcome measure, with so far limited psychometric evaluation in CSDH. This creates questions around its clinical significance. The implications of this can be seen in the interpretation of DEX-CSDH, an RCT of dexamethasone versus placebo, primarily in addition to surgery [18]. Whilst the trial showed a significant reduction of CSDH recurrence with dexamethasone, the increased adverse event profile and poorer functional outcomes have led to the recommendation that dexamethasone should not be used in the treatment of CSDH. The authors recommend that a patient with clinical symptoms of recurrence, that undergoes secondary intervention (i.e. repeat surgery) be used for recurrence criteria, in keeping with previously published trials [18, 40].

Trialists focused on recurrence rate will also need to be mindful of alternative emerging treatments. For example, results from RCTs evaluating pro-thrombotic agents, such as tranexamic acid are expected shortly [32], which if shown to be effective will have significant implications for how future trials evaluating MMAE should be run and interpreted.

### Limitations

The present meta-analysis has limitations. Firstly, although similar, all three trials had different selection criteria. There were also differences in baseline characteristics between the two treatment groups, in the inclusion and exclusion criteria. This was the main reason for employing random-effects meta-analysis. Because individual patient data was not accessible, it was not possible to pool this to examine if any differences were present. Secondly, the trials were all open label, due to the nature of the treatment, preventing a fully blinded outcome assessment, with different embolic agents. Thirdly, the primary outcome definitions included radiological progression, and recurrence or progression requiring repeat or rescue surgery respectively. Due to the limitations of the data, we were not able to extract data for surgical rescue or reoperation as a primary outcome. This is a significant limitation, given this is an empirical and comparable outcome. As random-effect assumes that variance occurs from study to study, we used this approach [12].

In the STEM trial, the follow up period was 180 days, compared to 90 days for both MAGIC-MT and EMBOLISE. We aimed to mitigate the effect of this difference by removing STEM in our sensitivity analysis, which showed similar results. MAGIC-MT and STEM excluded patients undergoing a craniotomy, who were included in the EMBOLISE trial. The effect of this difference on the study results is unclear. For STEM trial, the outcome data was also imputed, however this was according to the study protocol, with minimal difference compared to the absolute results.

In addition, it was not possible to stratify functional outcomes by surgical versus non-surgical management, as this data was not available in the full-text or supplementary material. There is a large amount of trial data that may be accessible to analyze in the future. Despite these limitations, the present review may help guide clinicians, and stimulate discussion regarding the efficacy of MMAE, particularly in surgical cohorts. We also did not analyze safety and adverse outcomes because each trial consistently demonstrated favorable safety profiles without any additional adverse safety outcomes.

## Conclusion

MMAE is an exciting new treatment that offers promise in CSDH; however, based on three RCTs data combined, there appears to be no significant difference in recurrence/progression rates, or functional outcome, when patients are categorized into those undergoing surgery, although benefit was seen in those undergoing nonsurgical treatment. The multiple ongoing trials identified by our systematic review will provide valuable additional data on these groups. The decision to use MMAE should be made on an individual basis, and requires further, focused trials with uniform inclusion criteria and outcome definitions, trials comparing embolic agents, antithrombotic/high risk of recurrence groups, cost-effectiveness, and functional outcomes, separating, rather than combining surgery and observation groups.

## Supplementary Information

Below is the link to the electronic supplementary material.

ESM 1 (DOCX 1.35 MB)

ESM 2 (DOCX 18.6 KB)

## Data Availability

No datasets were generated or analysed during the current study.
