# Supplementary material for: Middle meningeal artery embolization for chronic subdural hematoma: meta-analysis of three randomized controlled trials and review of ongoing trials
Source: Acta Neurochir (Wien). 2025 Jun 10;167(1):166. doi: 10.1007/s00701-025-06587-4 (PMC12152056; doi:10.1007/s00701-025-06587-4)

**Supplementary Figures**

Supplementary figure 1. Forest plot of overall primary outcome (pooled), with STEM trial removed.


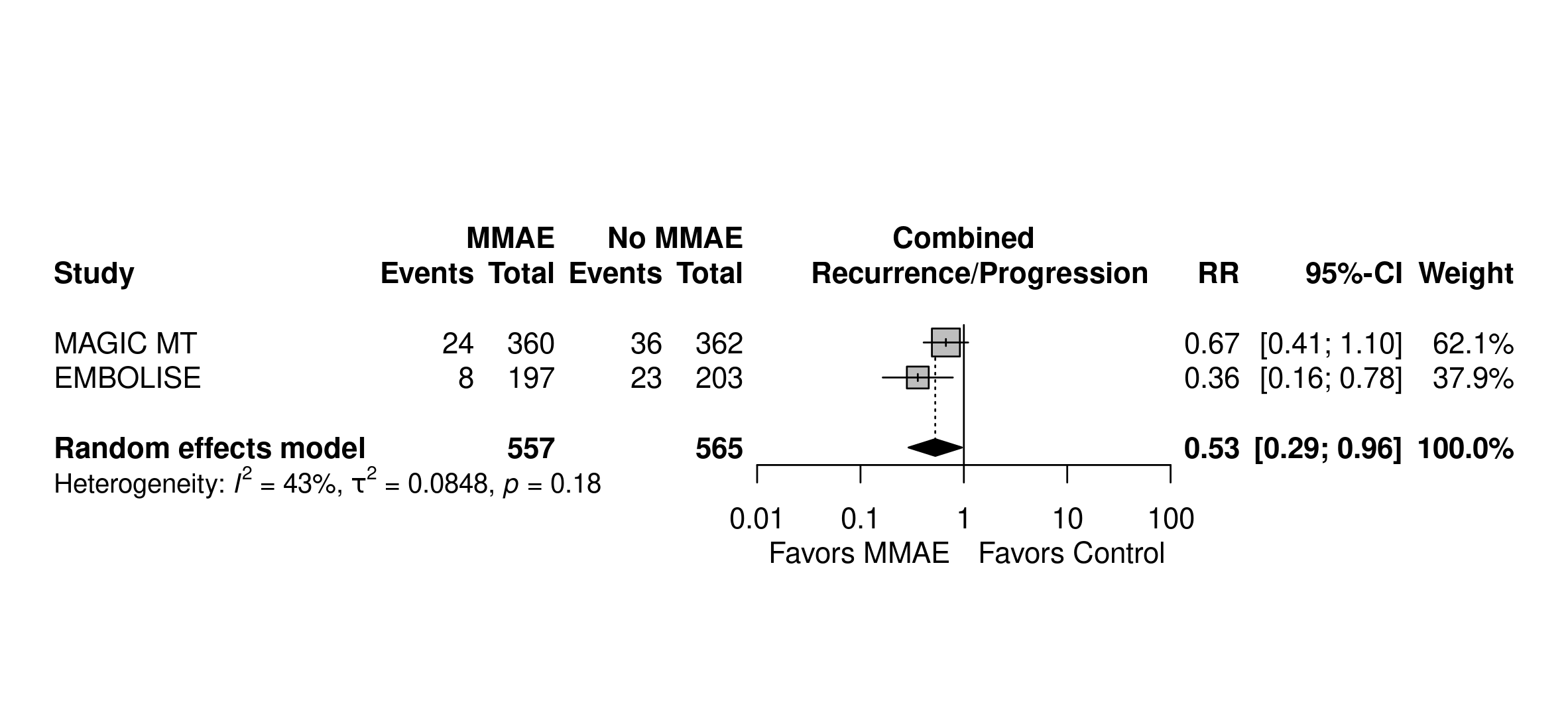


Supplementary figure 2. Forest plot of overall primary outcome (pooled) for patients undergoing surgery only, with STEM trial removed.


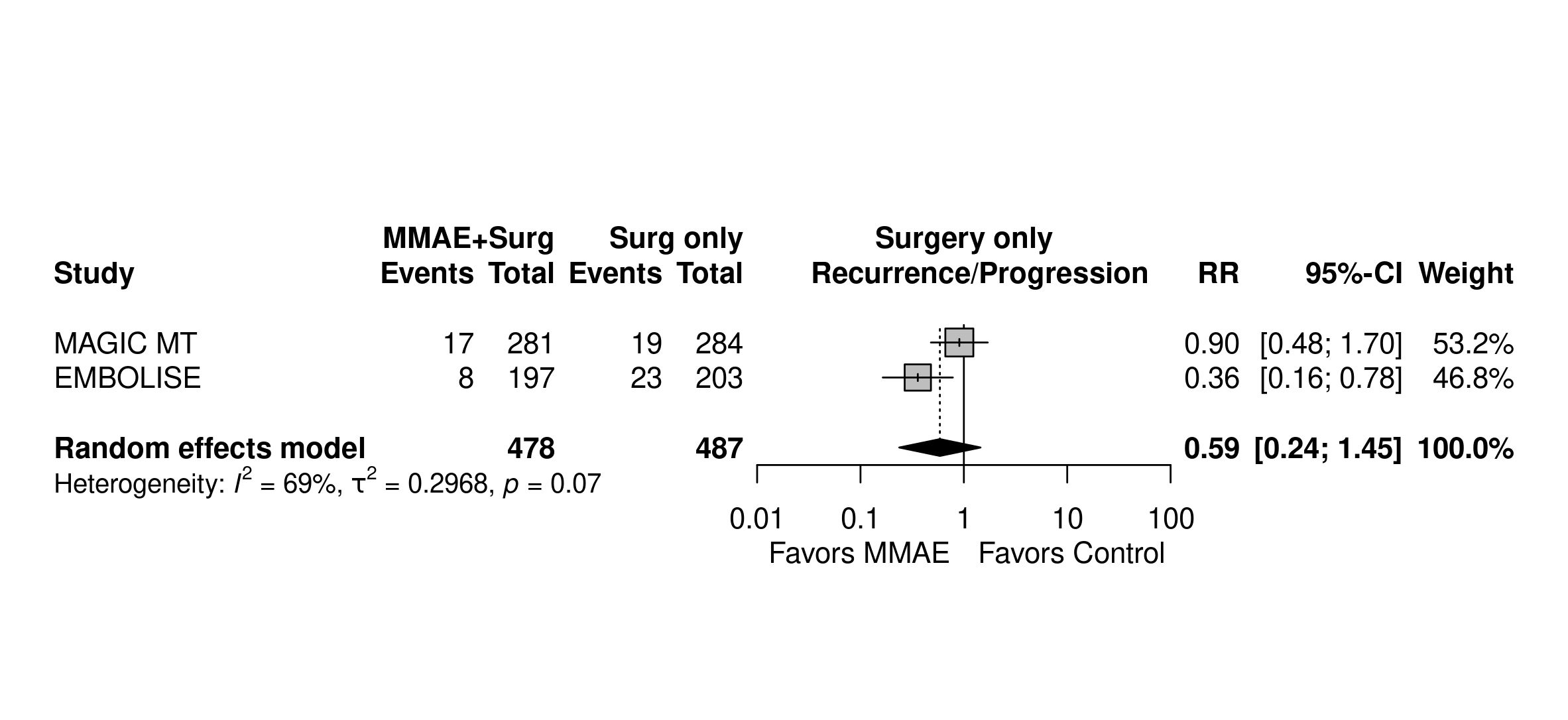


Supplementary Figure 3. Forest plot of overall primary outcome (pooled) as fixed effects only.


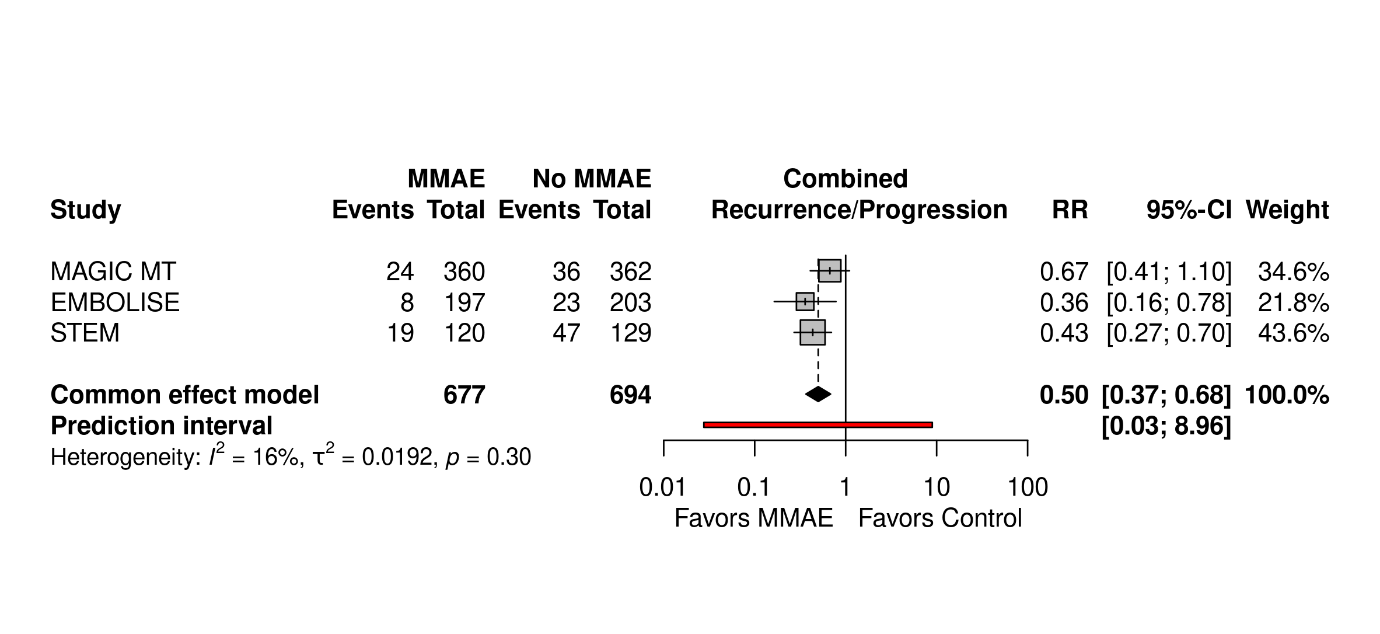


Supplementary Figure 4. Forest plot of overall primary outcome (undergoing surgical management only) as fixed effects only.


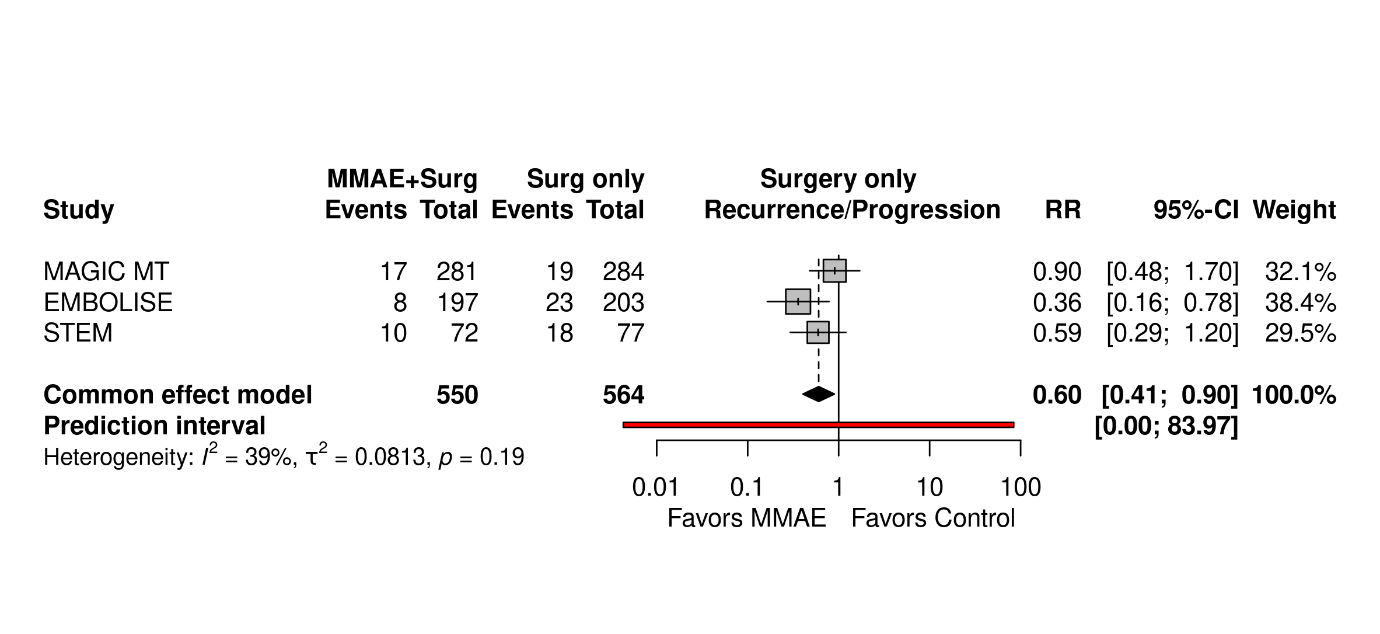


Supplementary Figure 5. Forest plot of overall primary outcome (undergoing nonsurgical management only) as fixed effects only.


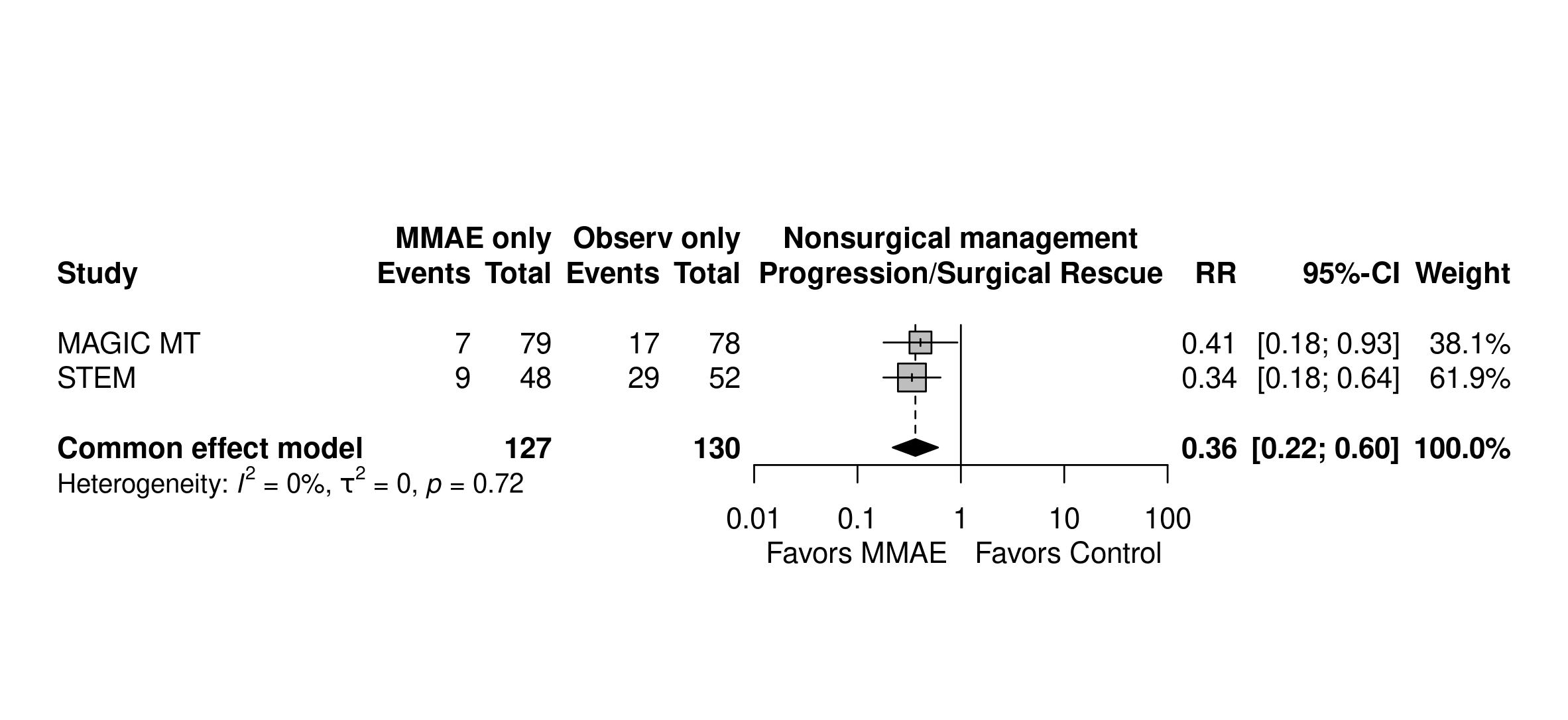


Supplementary Figure 6. Forest plot of functional outcome (mRS 0-2) as fixed effects only.


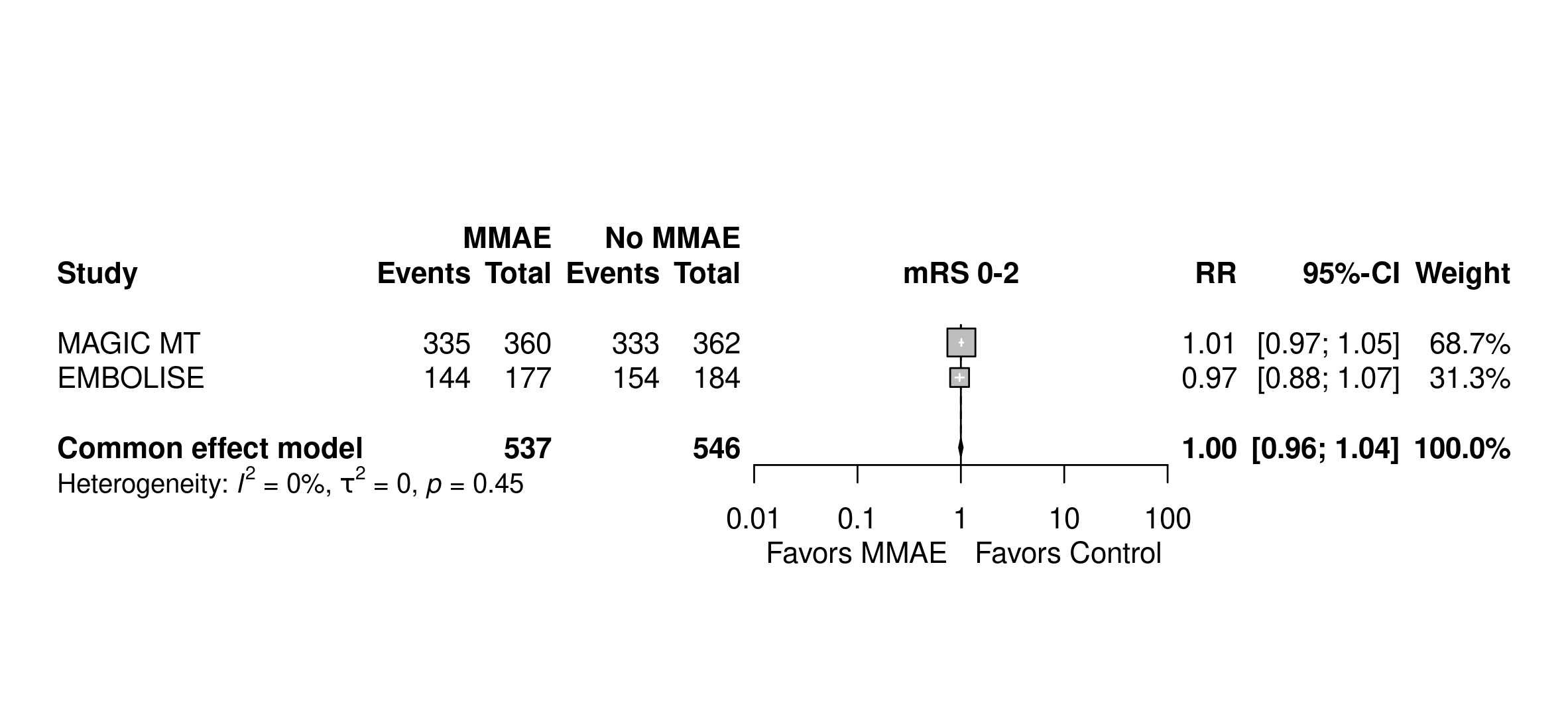


Supplementary Figure 7. Forest plot of functional outcome (mRS 0-3) as fixed effects only.


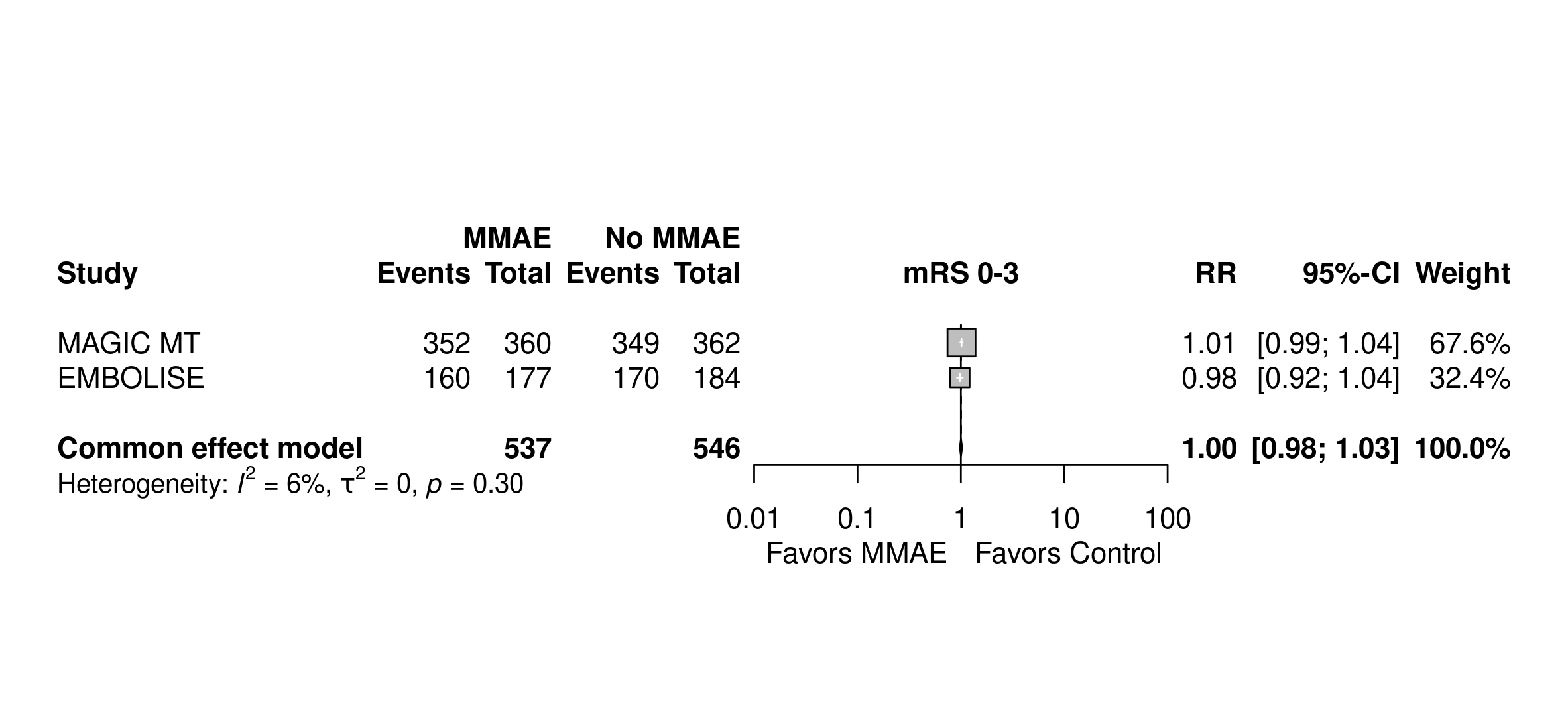


Supplementary Figure 8. Funnel plot for combined recurrence/progression for overall trial results.


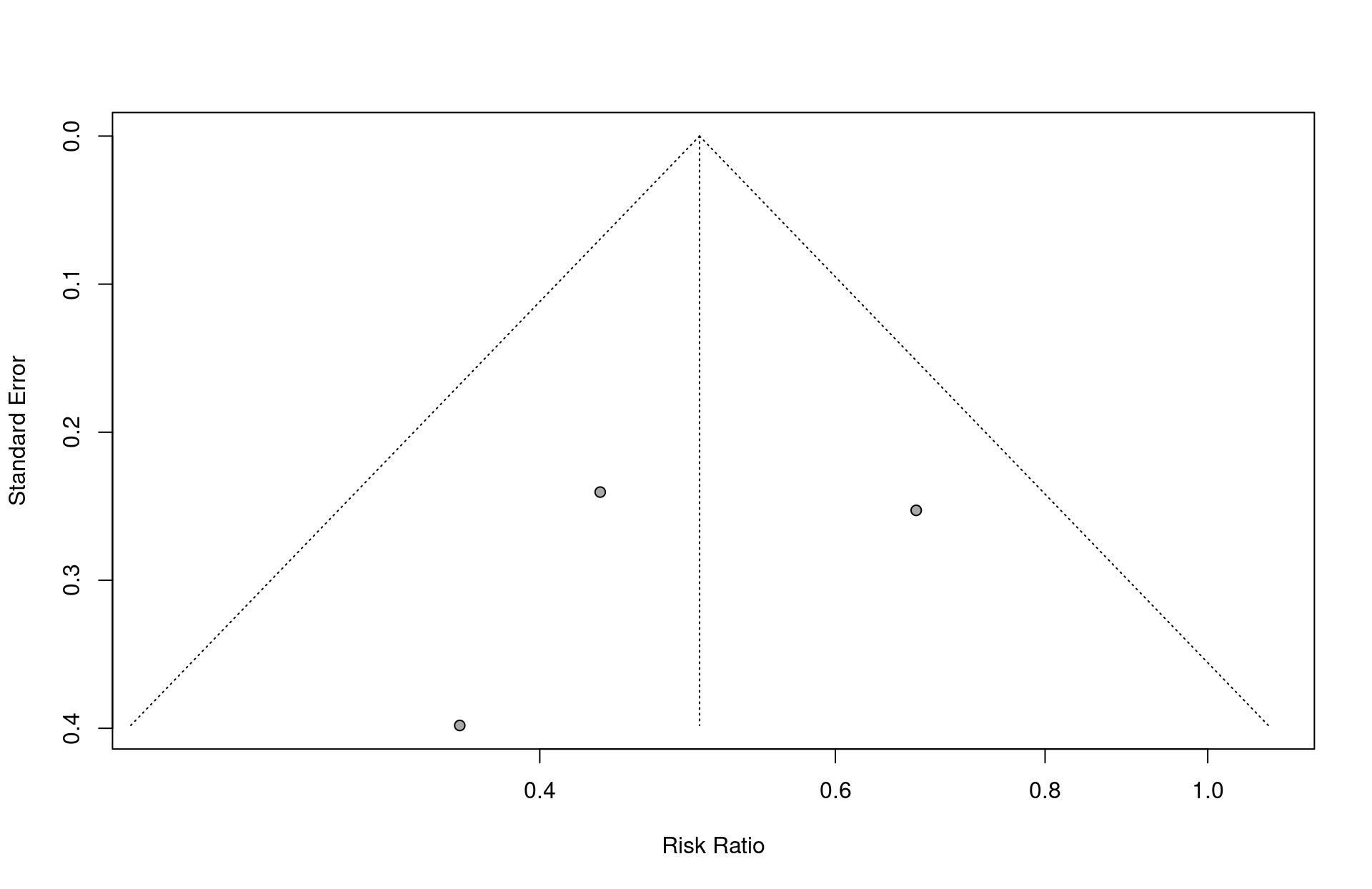


Supplementary Figure 9. Funnel plot for combined recurrence/progression for group undergoing surgery only.


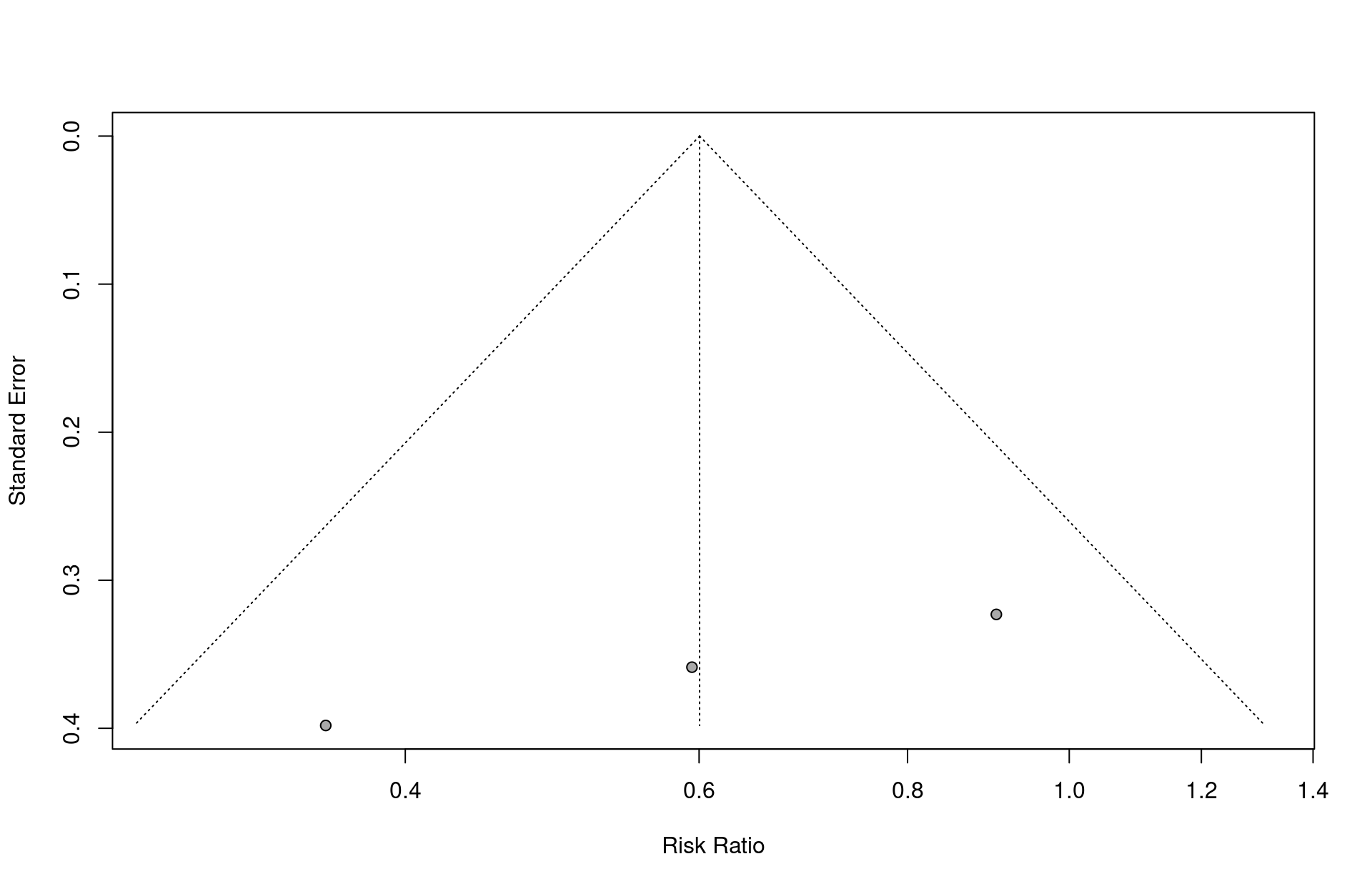


Supplementary Figure 10. Funnel plot for group undergoing surgery only, with reoperation as the primary endpoint.


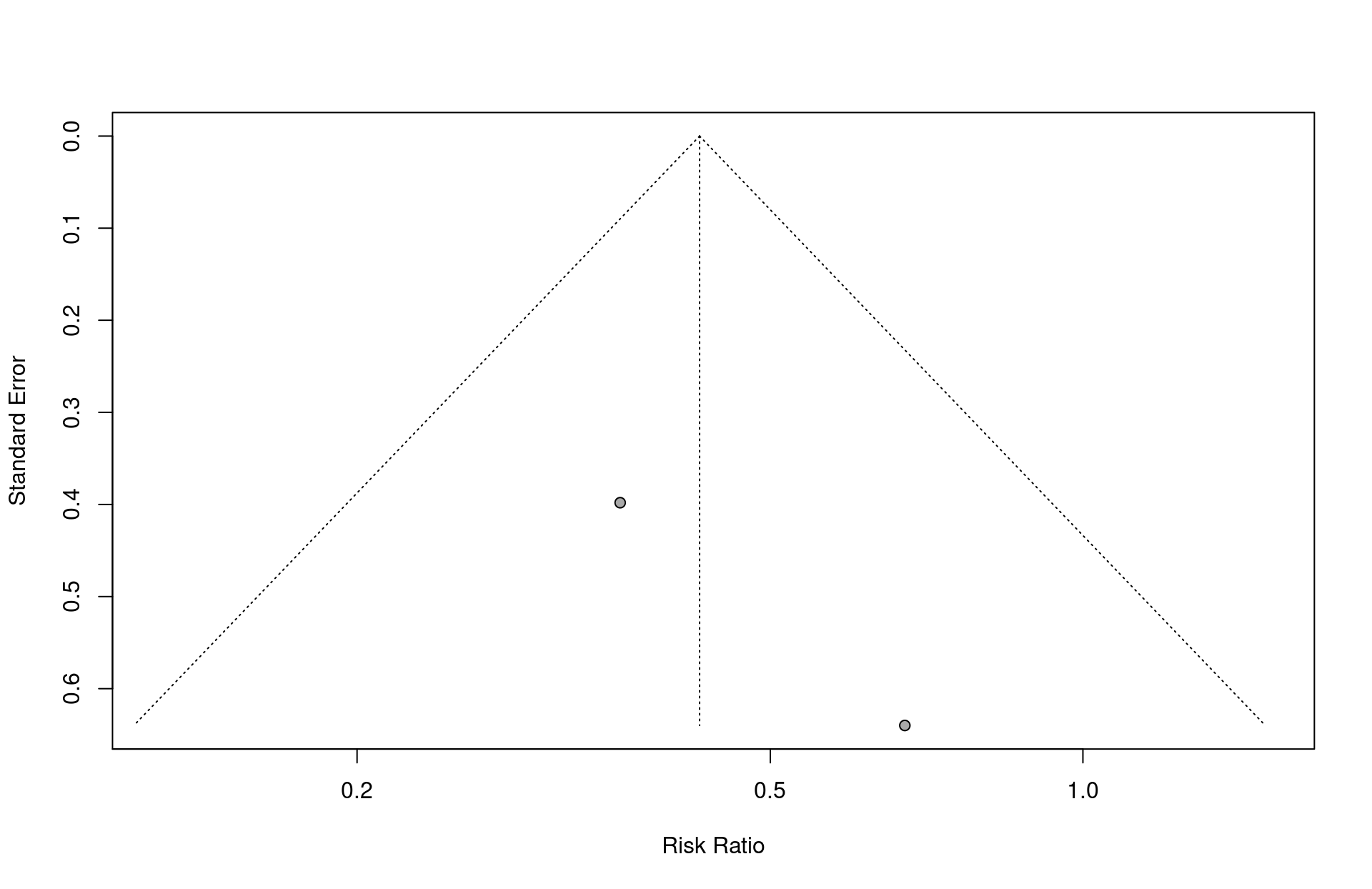


Supplementary Figure 11. Funnel plot for group undergoing nonsurgical management, with progression/surgical rescue as endpoint.


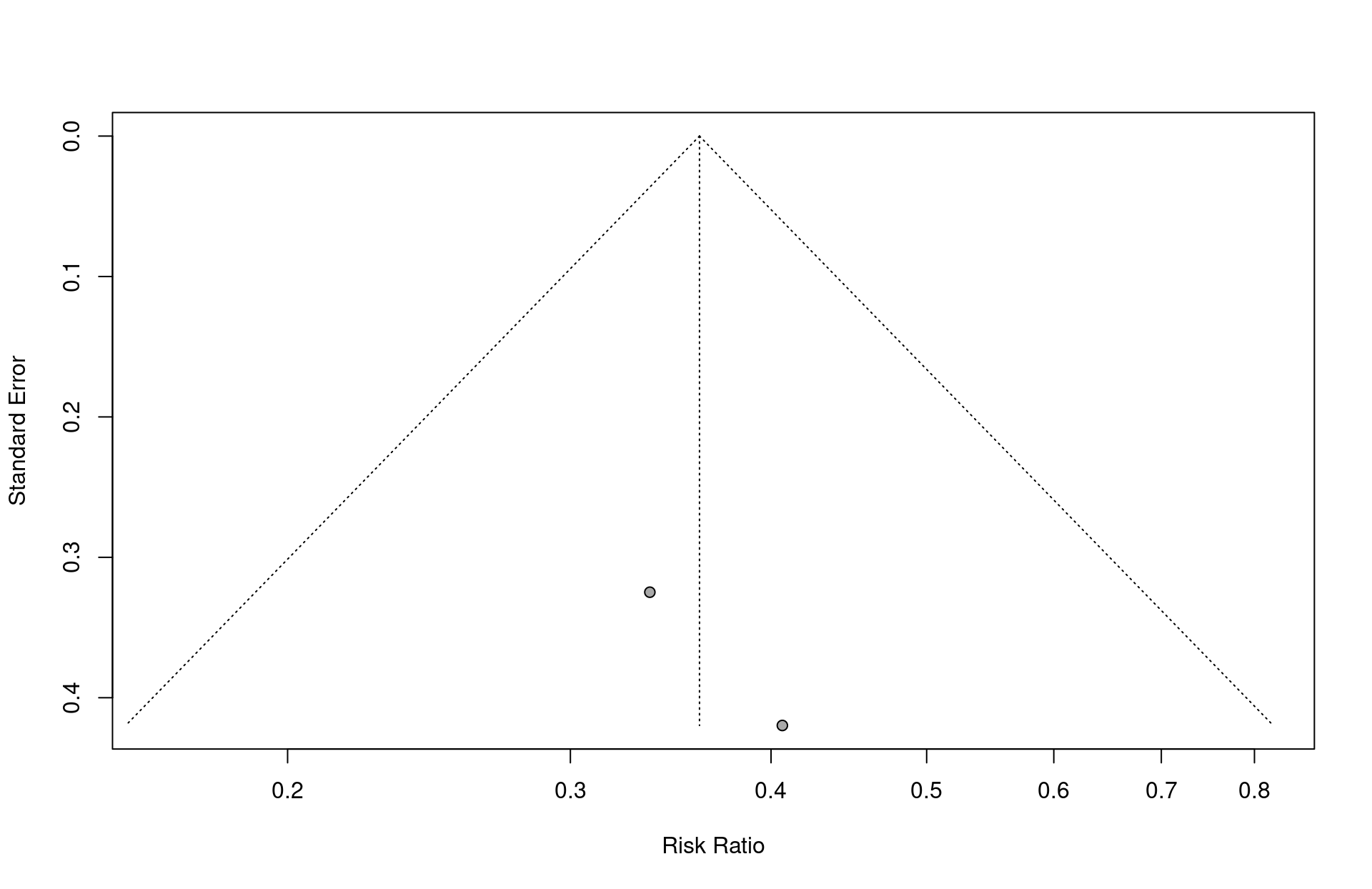


Supplementary Figure 12. Funnel plot for functional outcome (mRS 0-2).


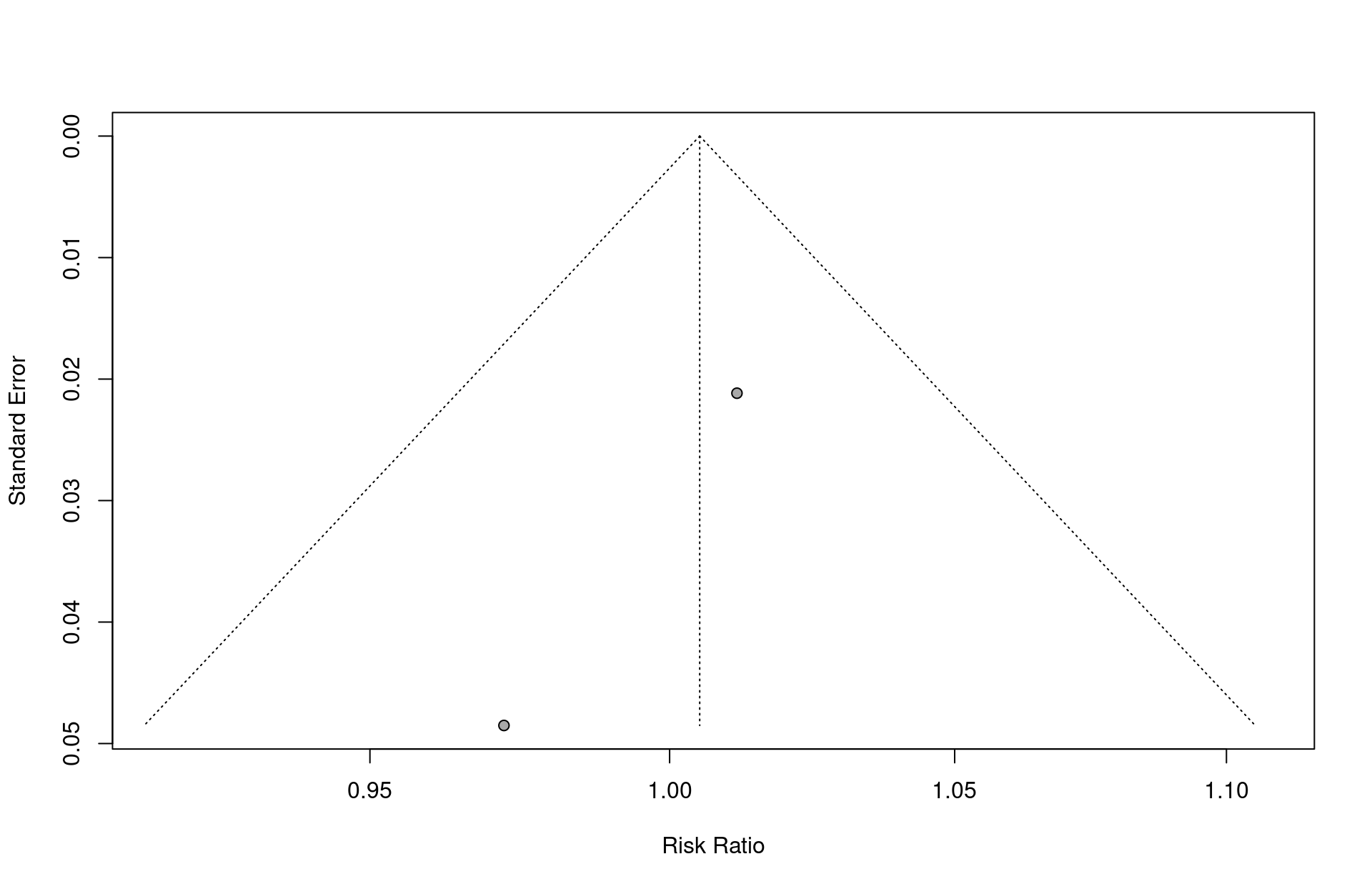


Supplementary Figure 13. Funnel plot for functional outcome (mRS 0-3).


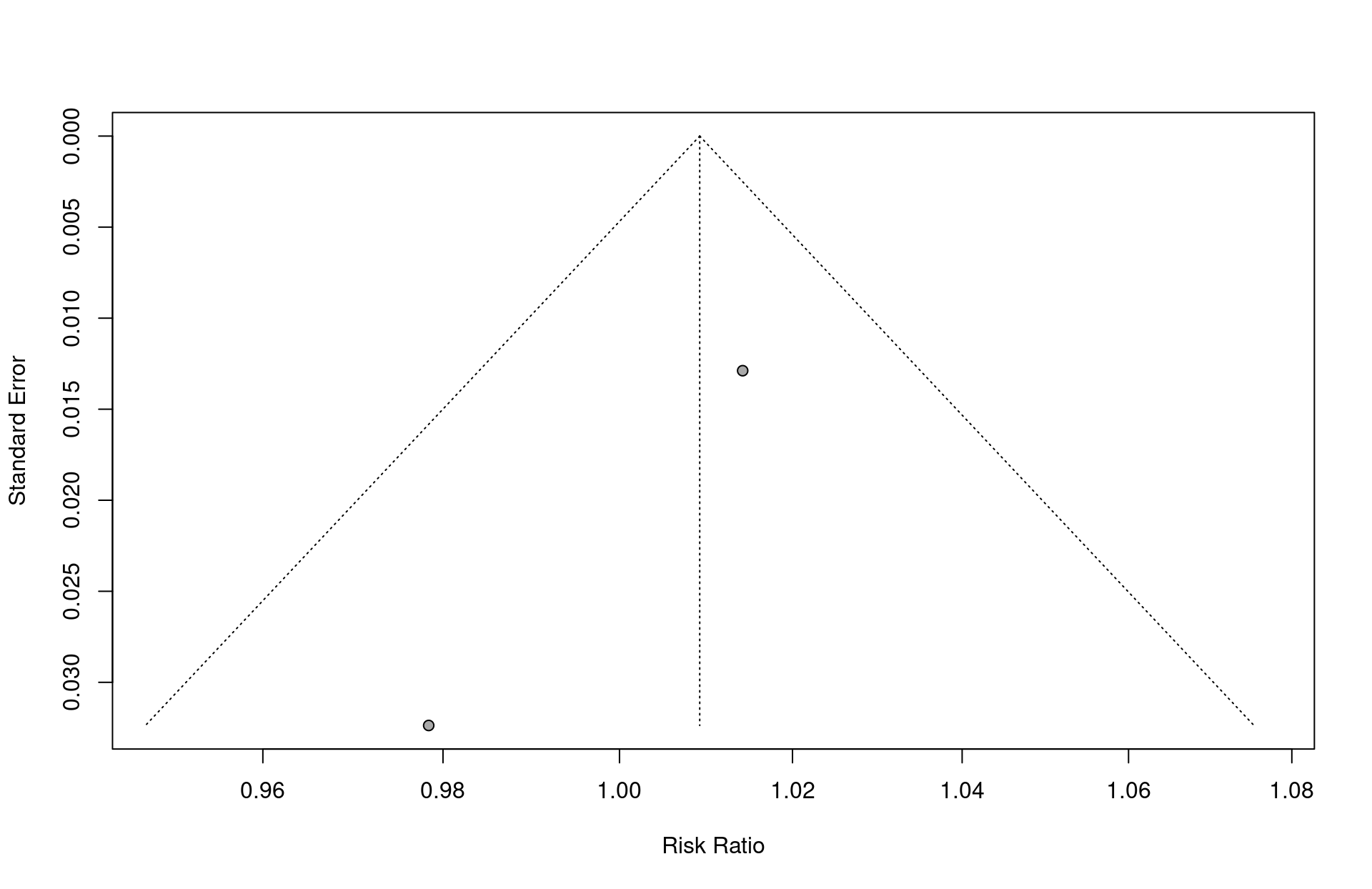


Supplementary figure 14. Forest plot of overall primary outcome (pooled), using Mantel-Haenszel modelling.


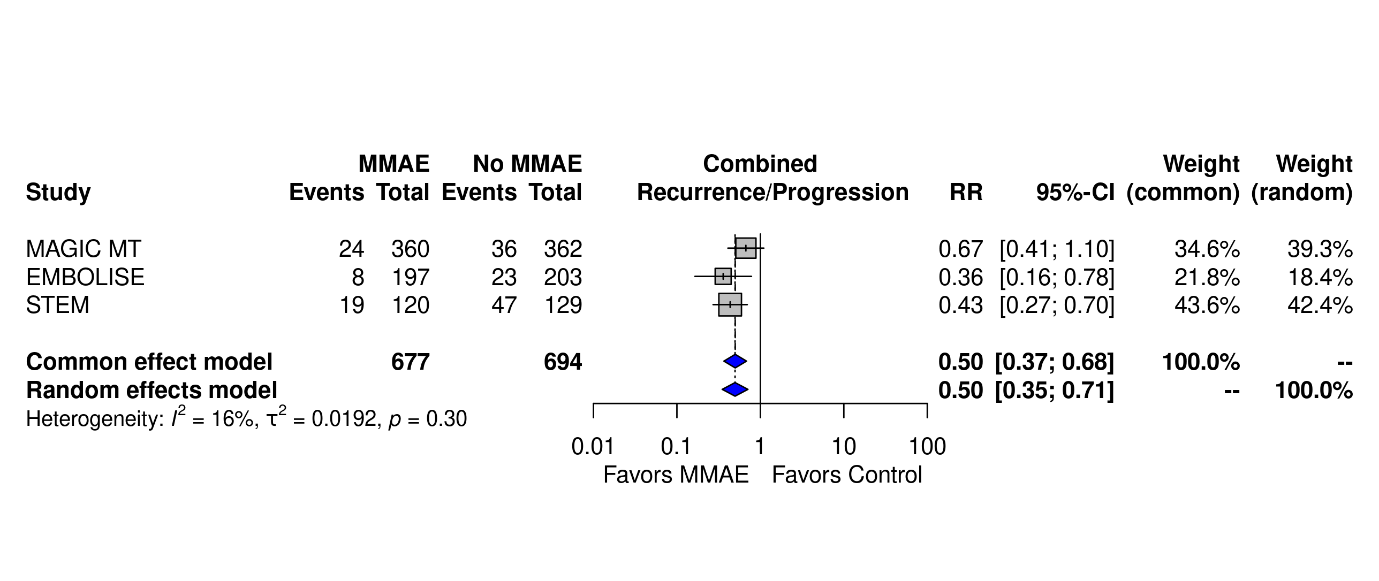


Supplementary figure 15. Forest plot of overall primary outcome (pooled) for patients undergoing surgery only using Mantel-Haenszel modelling.


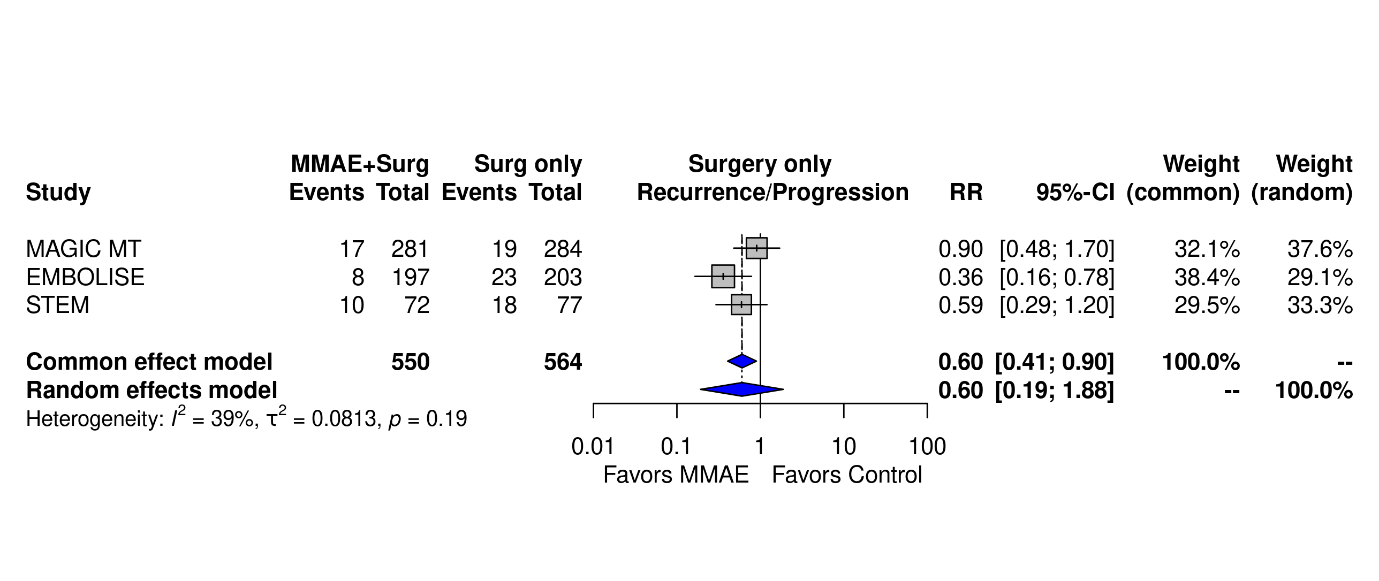


Supplementary figure 16. Forest plot of overall primary outcome (pooled) for patients undergoing surgery only, with reoperation as primary endpoint, using Mantel-Haenszel modelling.


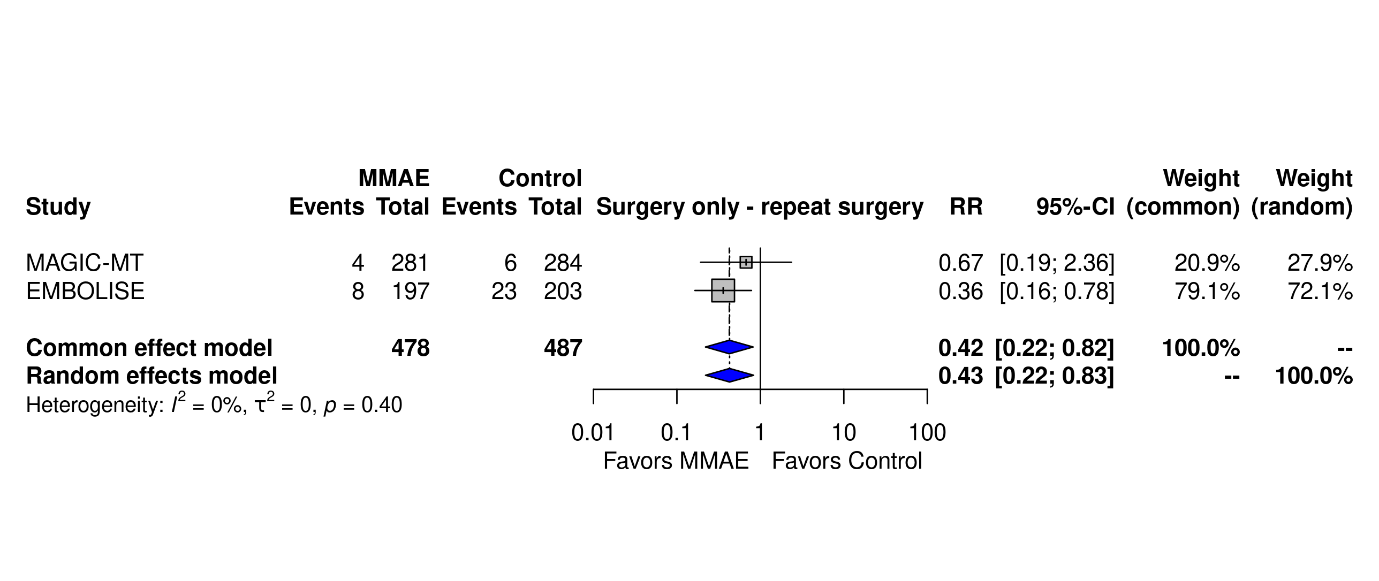


Supplementary Figure 17. Forest plot of overall primary outcome (undergoing nonsurgical management only) using Mantel-Haenszel modelling.


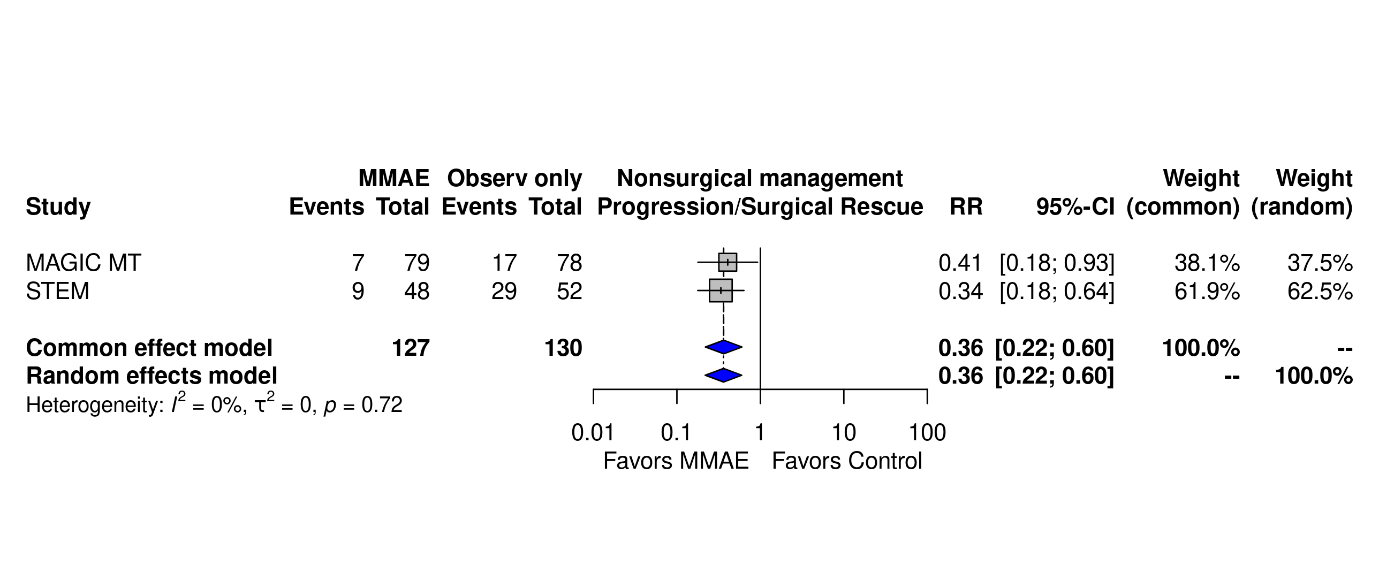


Supplementary Figure 18. Forest plot of functional outcome (mRS 0-2) using Mantel-Haenszel modelling.


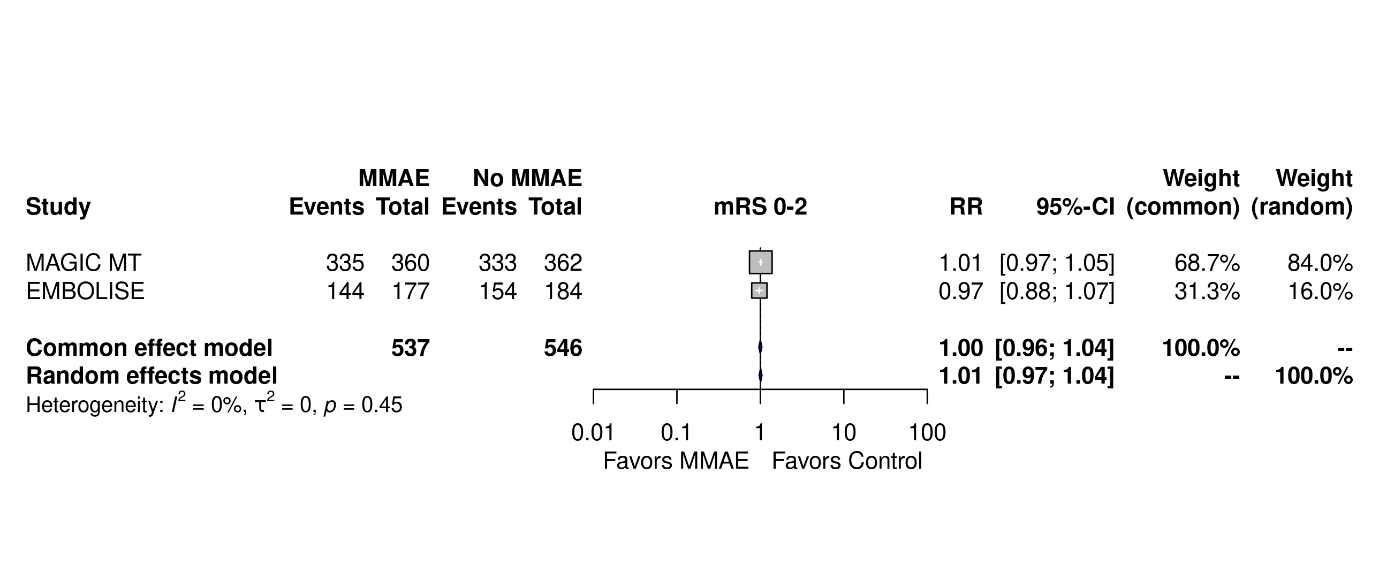


Supplementary Figure 19. Forest plot of functional outcome (mRS 0-2) using Mantel-Haenszel modelling.


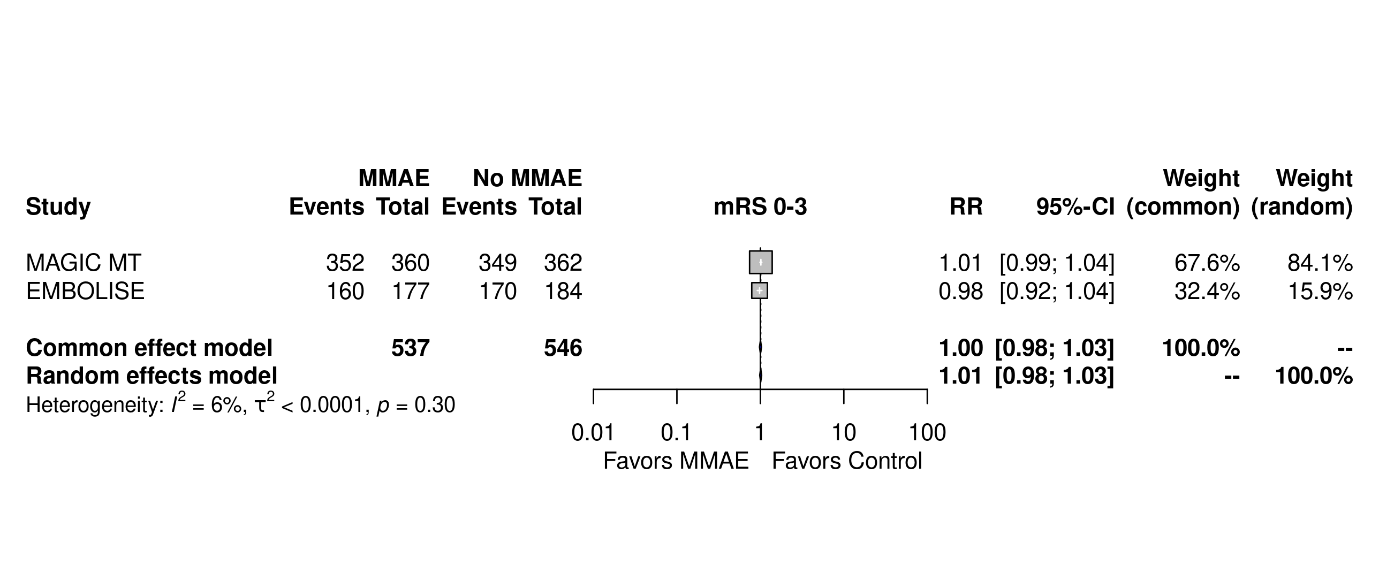


Supplementary Figure 20. Forest plot of overall primary outcome, using RevMan software.


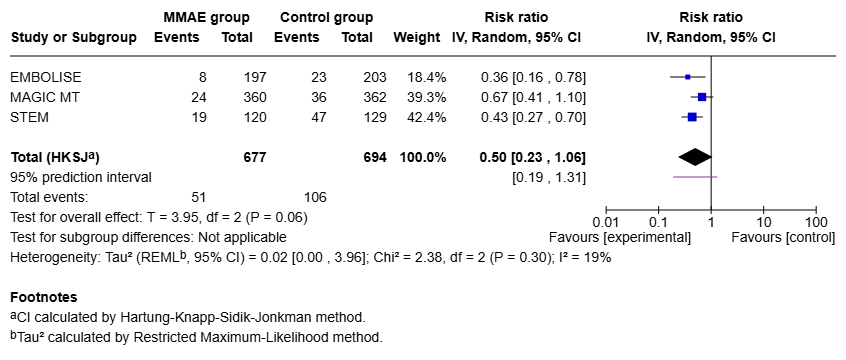


Supplementary Figure 21. Forest plot of overall primary outcome in group undergoing surgery only, using RevMan software.


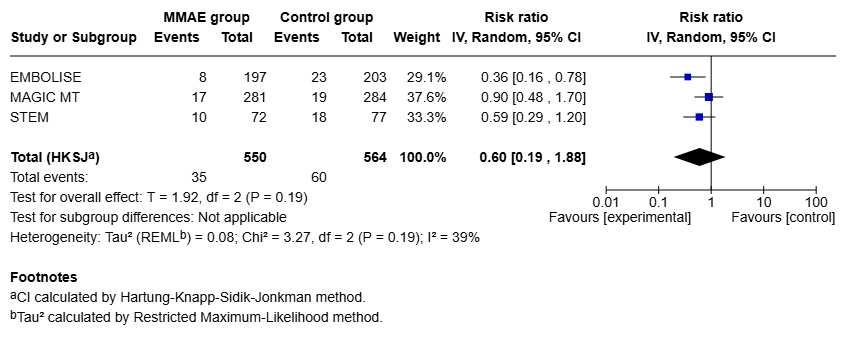


Supplementary Figure 22. Forest plot of overall primary outcome in group undergoing surgery only, with reoperation as primary endpoint, using RevMan software.


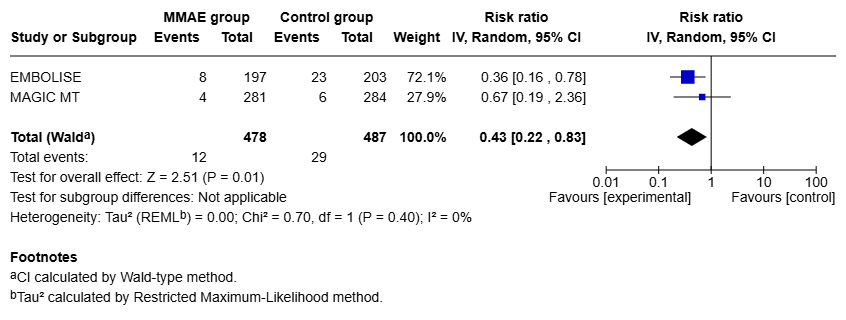


Supplementary Figure 22. Forest plot of overall primary outcome in group undergoing nonsurgical management only, using RevMan software.


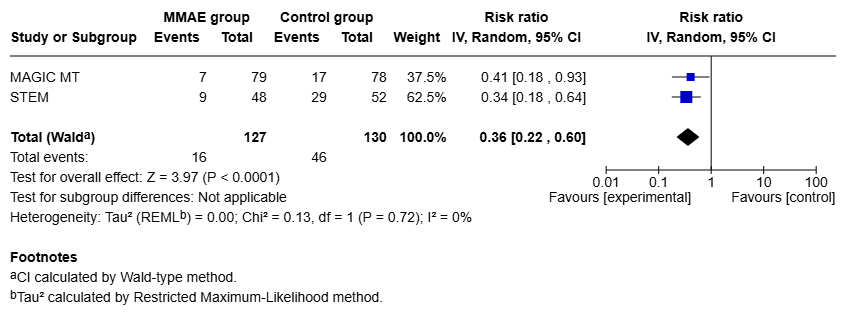


Supplementary Figure 23. Forest plot of mRS 0-2, using RevMan software.


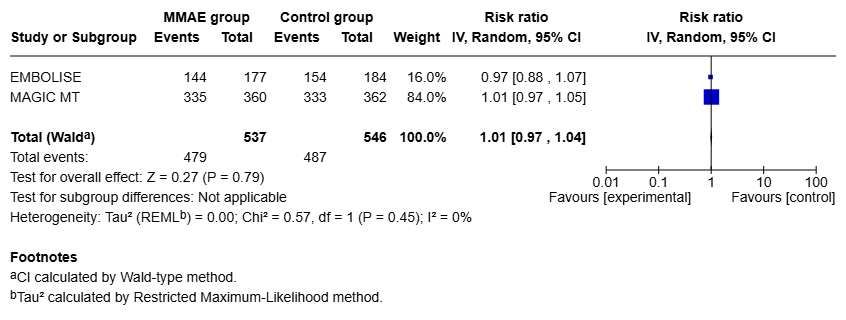


Supplementary Figure 24. Forest plot of mRS 0-3, using RevMan software.


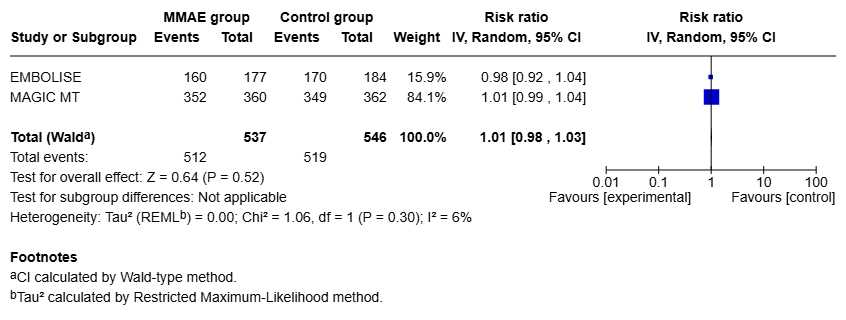

Supplement: Supplementary file 1 — (DOCX 1.35 MB) [file 701_2025_6587_MOESM1_ESM.docx]
