# Supplementary material for: Middle meningeal artery embolization for chronic subdural hematoma: meta-analysis of three randomized controlled trials and review of ongoing trials
Source: Acta Neurochir (Wien). 2025 Jun 10;167(1):166. doi: 10.1007/s00701-025-06587-4 (PMC12152056; doi:10.1007/s00701-025-06587-4)
Supplement: Supplementary file 2 — (DOCX 18.6 KB) [file 701_2025_6587_MOESM2_ESM.docx]

**Online supplementary tables**

**Supplementary table 1- List of trial registries included in literature search**

| Number | Database |
| --- | --- |
| 1 | Clinicaltrials.gov |
| 2 | WHO International Clinical Trials Registry Platform (ICTRP) |
| 3 | ISRCTN |
| 4 | Australian New Zealand Clinical Trials Registry (ANZCTR) |
| 5 | The Brazilian Registry of Clinical Trials (ReBEC) |
| 6 | Chinese Clinical Trial Registry (ChiCTR) |
| 7 | Korean The Clinical Research Information Service (CRIS) |
| 8 | The Clinical Trials Registry- India (CTRI) |
| 9 | Cuban Public Registry of Clinical Trials (RPCEC) |
| 10 | EU Clinical Trials Register (EU-CTR) |
| 11 | German Clinical Trials Register (DRKS) |
| 12 | Iranian Registry of Clinical Trials (IRCT) |
| 13 | International Traditional Medicine Clinical Trial Registry (ITMCTR) |
| 14 | Japan Registry for Clinical Trials (jRCT) |
| 15 | Lebanese Clinical Trial Registry (LBCTR) |
| 16 | Thai Clinical Trials Registry (TCTR) |
| 17 | Pan African Clinical Trials Registry (PACTR) |
| 18 | Registro Peruano de Ensayos Clínicos (REPEC) |
| 19 | Sri Lanka Clinical Trials Registry (SLCTR) |

**Supplementary Table 2- Databases and search terms used**

| **Clinicaltrials.gov** | Query | Results (14/05) |
| --- | --- | --- |
| 1 | Chronic subdural hematoma (condition) + Middle meningeal artery embolization (intervention) | 23 |
| **WHO ICTRP** | Query | Results (14/05) |
| 1 | Chronic subdural hematoma | 155 |
| ISRCTN | Query | Results (14/05) |
| 1 | Chronic subdural hematoma | 7 |
| AZNCTR | Query | Results (14/05) |
| 1 | Chronic subdural hematoma | 4 |
| REBEC | Query | Results (14/05) |
| 1 | Chronic subdural hematoma | 1 |
| **cHIctr** | Query | Results (14/05) |
| 1 | Chronic subdural hematoma | 4 |
| **CRiS** | Query | Results (14/05) |
| 1 | Chronic subdural hematoma | 1 |
| **CTRI** | Query | Results (14/05) |
| 1 | Chronic subdural hematoma | 8 |
| **RPCEC** | Query | Results (14/05) |
| 1 | Chronic subdural hematoma | 1 |
| **CTRI** | Query | Results (14/05) |
| 1 | Chronic subdural hematoma | 1 |
| **EU-CTR** | Query | Results (14/05) |
| 1 | Chronic subdural hematoma | 1 |
| **DKRS** | Query | Results (14/05) |
| 1 | Chronic subdural hematoma | 851 |
| **IRCT** | Query | Results (14/05) |
| 1 | Chronic subdural hematoma | 9 |
| **ITMCTR** | Query | Results (14/05) |
| 1 | Chronic subdural hematoma | 0 |
| **LBCTR** | Query | Results (14/05) |
| 1 | Chronic subdural hematoma | 0 |
| **TCTR** | Query | Results (14/05) |
| 1 | Chronic subdural hematoma | 1 |
| **PACTR** | Query | Results (14/05) |
| 1 | Chronic subdural hematoma | 3 |
| **REPEC** | Query | Results (14/05) |
| 1 | Chronic subdural hematoma | 3 |

**Supplementary Table 3. Reasons for exclusion after full-text assessment.**

| **Trail number/protocol number** | **Link** | **Reason for exclusion** |
| --- | --- | --- |
| NCT06274580 | <https://clinicaltrials.gov/study/NCT06274580> | Duplicate |
| TCTR20221014001 | <https://trialsearch.who.int/Trial2.aspx?TrialID=TCTR20221014001> | Prospective study |
| NCT04816591 | <https://classic.clinicaltrials.gov/ct2/show/NCT04816591> | Duplicate |
| NL-OMON27435 | <https://trialsearch.who.int/Trial2.aspx?TrialID=NL-OMON27435> | Duplicate |
| NCT04500795 | <https://clinicaltrials.gov/study/NCT04500795?cond=NCT04500795&rank=1> | Duplicate |
| NL-OMON55323 | <https://clinicaltrials.gov/study/NCT04511572> | Duplicate |
| NCT04095819 | <https://www.clinicaltrials.gov/study/NCT04095819> | Duplicate |
| NCT04750200 | <https://classic.clinicaltrials.gov/ct2/show/NCT04750200> | Duplicate |
| NCT06181994 | <https://clinicaltrials.gov/study/NCT06181994?cond=NCT06181994&rank=1> | Prospective cohort |
| JPRN-UMIN000051516 | <https://center6.umin.ac.jp/cgi-open-bin/ctr/ctr_view.cgi?recptno=R000058736> | Prospective cohort |
| NCT04574843 | <https://clinicaltrials.gov/study/NCT04574843> | Prospective cohort |
| NCT04500795 | <https://clinicaltrials.gov/study/NCT04500795> | Withdrawn |
| Emma-Can 2 | <https://clinicaltrials.gov/study/NCT04923984> | Prospective cohort |
| NCT03307395 | <https://clinicaltrials.gov/study/NCT03307395> | Prospective cohort |
| UMIN000028869 | <https://center6.umin.ac.jp/cgi-open-bin/ctr_e/ctr_view.cgi?recptno=R000033036> | Prospective cohort |
| U1111-1254-3825 | <https://www.anzctr.org.au/Trial/Registration/TrialReview.aspx?id=380090&isReview=true> | Not yet approved |

**Supplementary Table 4. Secondary outcomes**

| **Secondary outcome** | **Value (%)** |
| --- | --- |
| Cost effectiveness | 1 (4.3) |
| Length of stay | 6 (26.1) |
| Quality of life (Any measure) | 5 (21.7) |
| TDN score | 1 (4.3) |
| GOS (or GOS-E) | 2 (8.7) |
| Markwalder scale | 3 (13.0) |
| GCS | 1 (4.3) |
| Symptom improvement | 5 (21.7) |
| NIH Stroke scale | 5 (21.7) |
| MRS | 13 (56.5) |
| Hospital re-admission | 9 (39.1) |
| Midline shift or change | 3 (13.0) |
| Hematoma resolution | 10 (43.5) |
| Adverse events | 6 (26.1) |
| Mortality | 10 (43.5) |

**Supplementary Table 5. GRADE assessment of MMAE on primary outcome.**

| No of studies | Study design | Risk of bias | Inconsistency | Indirectness | Imprecision | Other considerations | Quality |
| --- | --- | --- | --- | --- | --- | --- | --- |
| 3 | Randomised trials | Not serious | Not serious | Differences in populations and outcome measures | Not serious | None | (⊕)(⊕)(⊕)(⊖)  Moderate |
